# Incidence, Temporal Trends, and Surgical Shift of Achilles Tendon Rupture: A Systematic Review and Meta-analysis

**DOI:** 10.1007/s40279-026-02397-5

**Published:** 2026-04-03

**Authors:** Roula Kotsifaki, Peter Malliaras, Christopher Byron, Joao Marques, Vasileios Korakakis

**Affiliations:** 1 Rehabilitation Department, Aspetar, Orthopaedic and Sports Medicine Hospital, Sports City Street, P.O. Box 29222, Doha, Qatar; 2 Department of Sports Medicine, Oslo Sports Trauma Research Center, Norwegian School of Sport Sciences, Oslo, Norway; 3 Physiotherapy Department, School of Primary and Allied Health Care, Monash University, Melbourne, VIC Australia; 4 Physiotherapy Program, Department of Health Sciences, School of Life Sciences and Health Sciences, University of Nicosia, Nicosia, Cyprus

## Abstract

**Background:**

Achilles tendon rupture is a severe injury affecting active and non-active populations, with considerable consequences on mobility, return to sport, and long-term function. Population-based incidence estimates provide important information for public health policy and resource allocation.

**Objective:**

This systematic review and meta-analysis aimed to estimate the global incidence of Achilles tendon rupture, evaluate temporal trends over six decades, and analyze subgroup differences by sex, age, region, and sport participation. Additionally, we examined trends in surgical treatment over time.

**Methods:**

A systematic review and meta-analysis was conducted in accordance with Preferred Reporting Items for Systematic review and Meta-Analyses (PRISMA) guidelines. Six databases (PubMed, CINAHL, Embase, Web of Science, Index Medicus, and Google Scholar) were searched through April 2025 to identify population-based studies reporting Achilles tendon rupture incidence. Only studies based on registries or representative samples were included. Random-effects models were used to pool incidence rates, and meta-regressions assessed time trends and subgroup differences. Incidence rates were calculated per 100,000 person-years and stratified by sex, age, country, geographic region, and sport participation. Surgical treatment trends were analyzed using quadratic meta-regression. Risk of bias was assessed using the Joanna Briggs Institute tool.

**Results:**

Twenty-eight studies reporting Achilles tendon ruptures from 1950 to 2022 were included, representing over 630 million individuals and 568,000 Achilles tendon rupture cases. The pooled global incidence was 15.7 per 100,000 person-years, spanning from 6.1 in 1979 to 31.1 per 100,000 person-years in 2021, with an average annual increase of 2.7% (95% confidence interval 2.0–3.3%). Male individuals exhibited a three-fold higher incidence than female individuals (pooled ratio 3.18; 95% confidence interval 2.50–4.04). Incidence peaked in male individuals aged 30–49 years (42.6 per 100,000 person-years) and in female individuals aged 40–49 years (17.2 per 100,000 person-years). Approximately 68% of Achilles tendon rupture were sport related. A significant shift in treatment patterns was observed: surgical repair rates peaked in 2003 and declined thereafter, indicating an increase in conservative treatment over the last 2 decades.

**Conclusions:**

The incidence of Achilles tendon rupture has risen steadily over the last six decades, with the greatest burden observed among middle-aged physically active men. A majority of ruptures are sport related, highlighting the need for preventive interventions. Surgical treatment has declined since 2003, reflecting shifts toward conservative management. These findings underscore the importance of continued surveillance, targeted prevention, and individualized treatment strategies to reduce the growing burden of Achilles tendon rupture worldwide.

**Clinical Trial Registration:**

PROSPERO registration ID: CRD420251020247.

**Supplementary Information:**

The online version contains supplementary material available at 10.1007/s40279-026-02397-5.

## Key Summary Points

**Table:** 

Achilles tendon rupture incidence has steadily increased over the past 60 years, with an average annual rise of 2.7%, and a peak pooled incidence of 31.1 per 100,000 person-years in the 2020s.
Male individuals are over three times more likely to experience Achilles tendon ruptures than female individuals (male-to-female ratio 3.18), with a peak incidence in men aged 30–49 years and in women aged 40–49 years.
Approximately 68% of Achilles tendon ruptures are associated with sport participation, especially ball and racquet sports.
Surgical repair rates peaked around 2003 and have since declined.

## Introduction

The Achilles tendon is the strongest and thickest tendon in the human body, yet it is the most frequently ruptured [1]. Its anatomical design, particularly its length, enhances movement efficiency by reducing energy cost and fatigue through energy storage and return [2]. Achilles tendon rupture (ATR) is a severe injury that compromises the tendon’s key role in locomotion. Although ATR most commonly affects physically active individuals engaged in sports involving sudden acceleration, deceleration, or jumping (e.g., basketball, volleyball, racket sports) [3, 4], it also occurs in sedentary and aging populations. This dual burden across athletic and non-athletic populations highlights ATR as a relevant and growing public health issue. Achilles tendon rupture can be managed via surgery or conservative treatment and trials have shown similar outcomes between these approaches [5, 6].

In recent years, the incidence of ATR has increased across several geographic regions and age groups [7–13]. This rise is likely driven by increased participation in recreational sports, higher physical activity levels among middle-aged adults, and improved awareness and diagnosis of tendon injuries. Despite this trend, much of the literature has focused on treatment [14], comparisons between surgical and conservative approaches [5, 15, 16], or rehabilitation protocols [17–19], rather than the broader epidemiology of ATR.

While country-level epidemiological studies have attempted to quantify the burden of ATR, and several reviews have tried to synthesize available evidence [12, 20–22], no study to date has conducted a formal meta-analysis or examined temporal trends in ATR incidence. Population-based surveillance is essential for accurately measuring the prevalence of ATRs, detect geographic or demographic disparities, and inform healthcare policy and injury prevention efforts. Stratified incidence estimates, by age, sex, sport participation, and country, are necessary to identify high-risk groups and allocate resources appropriately. Compared with other musculoskeletal injuries such as anterior cruciate ligament or hamstring injuries, ATR remains under-represented in large-scale surveillance systems. Effective injury prevention begins with establishing the incidence and severity of the problem, followed by identifying causes and mechanisms, steps that require robust and standardized surveillance [23], which is currently lacking for ATR.

Key questions remain unanswered: Are certain subgroups more vulnerable? Has incidence changed consistently over time? How do rates vary by country or geographic region? Addressing these questions is crucial for guiding injury prevention strategies, resource planning, and healthcare policy development. This study aims to fill these gaps by conducting a systematic review and meta-analysis of population-based studies to quantify ATR incidence globally, assess temporal trends, and explore subgroup differences by age, sex, region, sport participation, and treatment option.

## Methods

### Study Design

This meta-analysis was conducted in accordance with the recommendations of the Cochrane Collaboration [24], and is reported following the Preferred Reporting Items for Systematic review and Meta-Analyses (PRISMA) guidelines [25]. We systematically searched the published literature to identify incidence rates of ATR, without applying any date or language restrictions. The databases searched included PubMed, CINAHL, Embase, Web of Science, Index Medicus, and Google Scholar (screening the first 100 abstracts). The search was conducted up to 4 April, 2025. We used medical subject headings and text words adapted for each database covering three domains: “Achilles tendon,” “rupture,” and “epidemiology.” Full search methodology for each database is provided in Table S1 of the Electronic Supplementary Material (ESM). Reference lists of included studies were screened to identify any relevant articles not captured by the initial database search. References were managed using EndNote Version 20.4.

### Study Inclusion Criteria

Titles and abstracts of all references were screened independently and in duplicate by two reviewers (RK and VK) to identify original population-based studies reporting the incidence of ATR. As our objective was to examine trends in incidence rates over time, we applied strict inclusion criteria to ensure methodological rigor in the meta-analysis.

Studies were included if they met the following criteria: (1) original research; (2) peer reviewed; (3) population-based studies (i.e., studies covering nearly all members of the target population, such as registry data or sole-provider healthcare systems, or those using probability-based sampling methods); and (4) reported incidence of ATR or surgeries or provided raw data from which incidence rates could be calculated.

Studies were excluded if: (1) incidence was not reported or could not be calculated; (2) they focused exclusively on specific subgroups (e.g., only one sport, limited to specific age ranges such as > 60 years, military population); and (3) they were literature reviews, case studies, case series, conference abstracts, editorials, or letters to the editor. Disagreements regarding study inclusion were resolved through consensus.

### Risk of Bias Assessment

Two independent reviewers (RK and CB) assessed the risk of bias for all included studies using the Checklist for Prevalence Studies developed by the Joanna Briggs Institute [26]. This tool evaluates study quality across nine domains: (1) appropriate sampling frame; (2) appropriate sampling method; (3) adequate sample size; (4) detailed description of study subjects and setting; (5) sufficient coverage of the identified sample; (6) valid methods for identifying the condition; (7) standard and reliable measurement of the condition; (8) appropriate statistical analysis; and (9) adequate response rate.

### Data Extraction

To prevent duplicate counting of ATR cases, we excluded overlapping time periods from the same geographic regions. In cases of overlap, only one study was included, based on the following criteria applied in sequential order: (1) largest sample size (i.e., highest number of person-years of follow-up) and (2) most recent midpoint of the observation period. When studies reported on different data collection years or subgroups (e.g., by sex or age), all non-overlapping data were included.

Two authors (RK and JM) independently extracted the following data from each study: first author, publication year, study location (country and subnational region), study period, and incidence rate per 100,000 person-years. Stratified incidence data by age, sex, sport participation, and surgery incidence rates were extracted when available. Age-specific incidence was included if reported in decade intervals (e.g., 10–19, 20–29). Studies that reported broader age categories (e.g., 30–50 years) were excluded from age-specific meta-analyses and included only for qualitative analysis. For studies reporting in 5-year age groups, data were combined to create 10-year intervals.

If incidence rates were not directly reported, they were calculated using the formula: (cases × 100,000)/(total population × number of years). When incidence was reported separately for subgroups (e.g., male/female or sports/non-sports), data were combined to estimate the overall incidence rate. If incidence was reported for the overall study period without yearly breakdowns, the midpoint year of the study period was used to represent the estimate. Data presented only in graphical format were extracted using the WebPlotDigitiser (https://automeris.io/WebPlotDigitizer/).

Last, we extracted the proportion of sport-related ATRs as reported by the authors (or calculated it as sport-related cases ÷ total ATR cases × 100 when counts were provided). When studies listed multiple sport subcategories (e.g., team vs racket sports), we aggregated them into a single sport-related category for analysis.

### Statistical Analysis

In this comprehensive meta-analysis of ATR, a range of statistical methods were employed to synthesize incidence data, evaluate temporal trends, and analyze subgroup and treatment-related characteristics. Because of inconsistent reporting of confidence intervals (CIs) across studies, the Poisson rate function was used to calculate incidence rates per 100,000 person-years and corresponding 95% confidence intervals (CIs).

Meta-analyses were conducted only when at least two studies were available for a given outcome, using random-effects models with the restricted maximum likelihood estimator, which accounts for both within- and between-study variance. Incidence rates were log-transformed prior to pooling to improve distributional properties for the meta-analysis. Results were back-transformed and reported with 95% CIs for ease of interpretation. For proportions (e.g., percentage of sport-related injuries), logit transformations were used to stabilize variances before back-transformation to percentage values. Forest plots were generated to visually summarize these findings across studies.

For overall incidence rates, the midpoint of the study period was used as the reference year. Meta-regression subgroup analyses by sex, age, country, and World Health Organization-defined geographic regions [27] were conducted when sufficient data were available or included only for qualitative analysis.

Temporal trends in ATR incidence were examined using meta-regression models with year as continuous moderators, to provide a detailed estimation of changes over time. In addition, decade-specific analyses were performed to visualize broader changes over time using pooled incidence rates per decade. For studies spanning multiple decades, the data point closest to the midpoint of each decade was used.

To evaluate the temporal trend in ATR incidence rates for each study separately, we included studies reporting incidence over at least 5 years. Joinpoint regression models were applied to estimate the average annual percent change in incidence rates, assuming a Poisson distribution for the dependent variable and using year as the predictor.

To assess changes in surgical treatment over time, a random-effects meta-regression model with linear and quadratic year terms was used to capture non-linear patterns. Between-study heterogeneity was assessed using three standard statistics: Cochran’s *Q*-test (to test for significant variability), the *I*^2^ statistic (to quantify the proportion of variability because of heterogeneity), and tau-squared (*τ*^2^) [to estimate between-study variance].

All statistical analyses were performed in R (version 2024.12.1) using the metafor, meta, and ggplot2 packages. Statistical significance was set at *p* < 0.05. This study was registered at the International Prospective Register of Ongoing Systematic Reviews (PROSPERO) under CRD420251020247.

## Results

### Study Characteristics

**Fig. 1:**
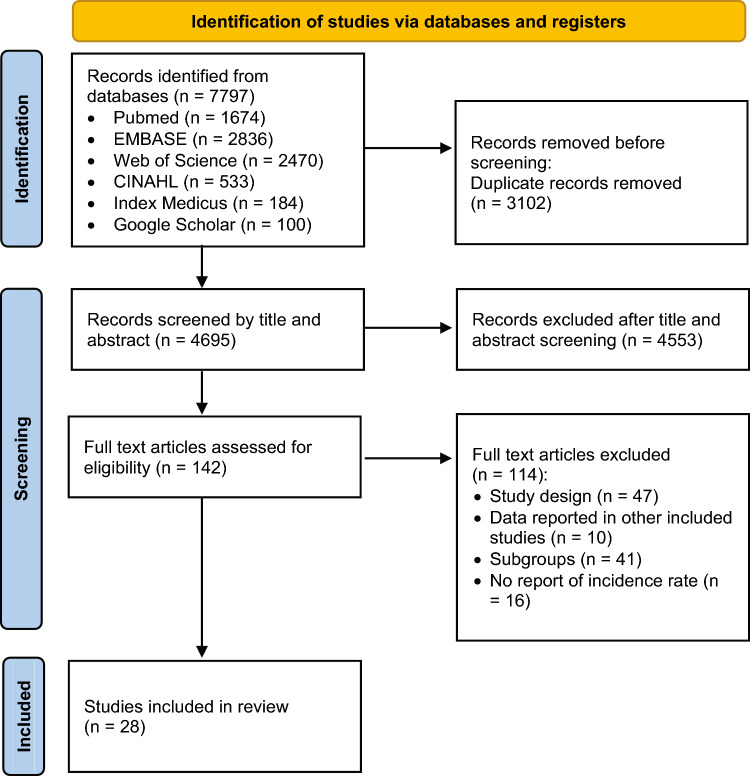
Preferred Reporting Items for Systematic reviews and Meta-Analyses (PRISMA) study selection flowchart

The electronic search yielded a total of 7797 potentially relevant articles. After removing duplicates, 4695 articles were screened by title and abstract. The full texts of 144 articles were assessed for eligibility based on the predefined inclusion/exclusion criteria. Finally, 28 articles [7–13, 22, 28–47] met the inclusion criteria for this review. No additional articles were identified through hand searching (Fig. 1).

A total of 28 studies were included in the meta-analysis [7–13, 22, 28–47]. Three of these reported incidence rates on Achilles tendon repair [35, 40, 43]. Two studies reported different outcomes from the same population [36, 37]. For one study [43], we extracted results only for years 2002–8 as it was part of another study [41].

Collectively, the included studies represented a population of over 630 million individuals and reported 562,868 ATRs. Eighteen studies were conducted in Europe, five in the Americas, two in Oceania, and three in Asia. The data collection periods spanned from 1950 to 2022, with a median follow-up duration of 11.5 years across studies. The demographic details of the included studies are summarized in Table 1.

**Table 1:** Demographics of included studies

Author, year	City, region, country	Healthcare setting	Population	Duration	Participant count	Participant age	Peak age IR	Patients operated	Sport related ruptures	Seasonality
Nillius, 1976 [28]	Malmo, Sweden	1 hospital serves the whole city	230,000^a^	1950–73	Total: 229 Male: 200 Female: 29	Not reported	40–49 y (all)	229 (100%)	134 (58.5%) Male: 121 Female: 13	
Moller, 1996 [9]	Malmo, Sweden	1 hospital serves the whole city	230,000	1987–91	Total: 153 Male: 132 Female: 21	Male: mean 42 y (range: 20–80 y) Female: 52 y (range: 25–80)	30–39 y (all)	Not reported	98 (64.1%) Male: 91 Female: 7	
Levi, 1997 [29]	Copenhagen, Denmark	Municipality of Frederiksberg	88,000	1978–95	Total: 213 Male: 158 Female: 55	All: median: 41 y (range: 18–86)	Male: 30–34 y Female: 30–34 y	Not reported	172 (80.6%) Only total reported	
Houshian, 1998 [30]	Ribe County, Denmark	5 hospitals in Ribe County, database	220,000	1984–96	Total: 718 Male: 544 Female: 174	All: mean 42.1 y (range: 3–82) y	Sports: 30–49 y Non-sports: 50–59 y	643 (89.6%)	533 (73%) Male: 407 Female: 126	
Maffulli, 1999 [8]	Scotland	National, database, surgical, not private visits	5,100,000	1980–95	Total: 4201 Male: 2628 Female: 1573	Not reported	Male: 30–39 y Female: 80 + y	Almost all are likely to undergo surgery	Not reported	No evidence of seasonality, trend for lower IR in autumn
Suchak, 2005 [22]	Edmonton, Alberta, Canada	Acute care hospitals in Edmonton	940,000	1998–2002	Total: 394 Male: 313 Female: 78	All: mean 41.4 y (range: 13–79 y)	Male: 30–39 y Female: 40–49 y	Not reported	296 (75%)	No evidence of seasonality, trend for lower IR in autumn and more in spring
Sode, 2007 [31]	Funen County, Denmark	Database of Funen County	470,000	1991–2002	Total: 1538 Male: 1154 Female: 384	All: mean age: 44.2 y	All: 30–49 y	Not reported	Not reported	
Tumilty, 2007 [12]	New Zealand	National	4,000,000	Jul 1998- Jun 2003	Total: 412 (2003) Male: 241 Female: 171	Not reported	All: 35–44 y (2003) Male: 40–44 y Female: 35–39 y	Not reported	233 (56.6%) (2003)	
Clayton, 2008 [32]	Edinburgh, Scotland	Single site (Edinburgh Orthopaedic Trauma Unit)	535,000	1996–2000	Total: 298 Male: 203 Female: 95	Male: mean 43.2 y Female: mean 46.9 y	Male: 30–49 y Female: 40–49 y	Not reported	Not reported	
Nyyssönen, 2008 [10]	Finland	National, database, patients admitted to hospital, outpatients were excluded	5,200,000 (1999)	1987–99	Total: 7375 Male: 5826 Female: 1549	Mean 42 ± 12 Male: mean 41.7 Female: mean 44.0	All: 39–40 y	Probably all *(“conservative treatment was very rare during 1987 and 1999 in Finland”)*	Not reported	No evidence of seasonality
Gwynne-Jones, 2011 [33]	Dunedin, New Zealand	Consecutive patients in the only university hospital in a city Inclusion: complete, traumatic, closed, 15–60 y, living in the area	177,000	Jul 1999-Feb 2008	Total: 363 Male: 197 Female: 166	Male: mean 41.2 ± 9.6 Female: mean 37.6 ± 9.3 15–60 y (eligibility criteria)	All: 41–50 y	143 (39.4%)	285 (78.5%)	
Lantto, 2015 [34]	Oulu, Finland	University hospital and 4 private clinics	94,000 (1979), 142,000 (2011)	1979–2011	Total: 515 Male: 456 Female: 59	All: mean 43 ± 13 (19–90) Male: mean 42 ± 13 (19–79) Female: mean 46 ± 15 (23–90)	Male: 30–39 y Female: 40–49 y	429 (83.3%)	371 (70%)	
Mattila, 2015 [35]	Finland	National, registry, only surgeries	5,200,000^a^	1987–2011	Total: 15,252 (only surgeries)	Male: median 41 y (18–90) Female: median 45 y (18–91)	Male: 40–59 y Female: 40–59 y	15,252 (only surgeries)	Not reported	
Ganestam, 2016 [7]	Denmark	National, database	5,400,000	1994–2013	Total: 33,160 Male: 24,939 Female: 8221	1994 median (range): Male: 42 y (6–84) Female: 42 y (2–93) 2013 median (range): Male: 49 y (6–98) Female: 48 y (7–93)	All: 41 y	Not mentioned (changed over the years)	Not reported	Lower IR in summer and more in autumn
Sheth, 2017 [36]	Ontario, Canada	Emergency department, database, only surgical, acute, closed, index, > 18 y	13,000,000	Apr 2002- Mar 2014	Total: 29,531 Male: 19,609 Female: 9922	All: median 44 y (IQR 26–62)	Not reported (only average for surgical patients)	Overall: 4562 (15.5%) 2002:21.5% 2010: 20.6% 2014: 6.5%	Not reported	
Sheth, 2017 [37]	Ontario, Canada	Emergency department, database, closed, index, > 18 y	13,000,000	2003–13	Total: 27,607 Male: 18,364 Female: 9243	All: median 44 y (IQR 18)	Male: 30–39 y Female: 40–49 y		Not reported	Lower IR in winter and statistically significant more in summer
Yasui, 2017 [38]	USA	Insurance database (9% of the USA population aged < 65 y is represented)	20,086,126	2007–11	Total: 21,305	Not reported	All: 30–39 y	Not reported	Not reported	
Lemme, 2018 [39]	USA	National Electronic Injury Surveillance System (NEISS) database (emergency departments from 100 hospitals)	320,000,000	2012–16	Total: 32,906 Male: 25,374 Female: 7533	All: mean 37.5 ± 13.9 Male: mean 38.0 ± 13.9 Female: mean 36.2 ± 16.9	Male: 20–39 y Female: 40–59 y	Not reported	26,950 (81.9%)	
Longo, 2020 [40]	Italy	National, database hospital discharge, only surgeries	60,300,000	2001–15	Total: 118,652 Male: 98,376 Female: 20,271	Not reported	Male: 35–44 y Female: 35–44 y	118,652 (only surgeries)	Not reported	
Park, 2021 [41]	South Korea	National, insurance database covering 97.2% of the total population	50,000,000^a^	2009–17	Total: 112,350	Not reported	All: 41–50 y	44,248 (39.4%)	Not reported	Lower IR in winter and more in summer
Yamaguchi, 2021 [13]	Japan	National, insurance database (contains almost all inpatient and outpatient medical claims), primary, acute, > 20 y	127,000,000^a^	Apr 2010- Mar 2017	Total: 112,601 Male: 75,334 Female: 37,267	All: median 45–49 y	All: 40–59 y	79,033 (70.2%)	Not reported	
Leino, 2022 [42]	Finland	National, database, all inpatient and outpatient, acute, > 16 y	5,200,000^a^	1997–2019	Total: 30,162 Male: 22,735 Female: 7427	Operatively: 44 ± 12.6 (16–91) Non-operatively: 53 ± 16.3 (16–101)	All: 30–49 y	13,242 (44%)	7279 (55%) of surgical cases	
Park, 2022 [43]	South Korea	National, database covering 97.2% of the total population, only index surgeries	713,456	2002–15	Total: 850 Male: 653 Female: 197	20 y to ≥ 80 y	Male: 30–39 y Female: 30–39 y	850 (only surgeries)	Not reported	
Carmont, 2023 [44]	East Shropshire, UK	Single hospital, medical records	250,000	Feb 2009-Aug 2022	Total: 436	All: mean 48 ± 14	All: 40–49 y	Not mentioned	218 (50.1%)	Lower IR in winter and more in summer
Cretnik, 2023 [45]	Maribor, Slovenia	University clinical center, only center for ATR in Maribor, index, closed, complete, mid-portion, > 18 y	273,485	1991–2015	Total: 524 Male: 486 Female: 38	All: mean 39 ± 10.9 (range 20–83)	Not reported	Not mentioned	356 (67.94%)	
Maempel, 2023 [46]	Lothian, Scotland	Regional, database, primary ATR	858,090	2011–16	Total: 783 Male: 567 Female: 216	Male: median 48 y (IQR 38–60) Female: median 48y (IQR 41–65)	All: 40–49 y Male: 40–49 y Female: 40–49 y	Not mentioned	388 of 772 (50.3%)	Lower IR in autumn and more in summer
Briggs-Price, 2024 [47]	Leicester, UK	Single emergency department, medical records	540,000^a^	2016–20	Total: 361 Male: 286 Female: 75	All: median: 45 y (IQR: 19)	Not reported	10 (2.9%)	236 (65.2%)	
Svedman, 2024 [11]	Sweden	National, registry, > 18 y	10,000,000	2002–21	Total: 53,688 Male: 42,140 Female: 11,548	2002: median 44 y (CI 43.8–44.2) 2021: median 50 y (CI 49.7–50.3)	Male: 40–49 y Female: 40–49 y	15,045 (28%)	Not reported	

*CI* confidence interval, *IQR* interquartile range, *IR* incidence rate, *y* years

^a^Population estimation from https://www.macrotrends.net/

The quality assessment for each individual study is provided in Table S2 of the ESM. One item from the checklist (response rate) was deemed not applicable, as all included studies were epidemiological in nature and primarily relied on registry or administrative data.

### Overall and Geographic Incidence Rates of ATR

**Fig. 2:**
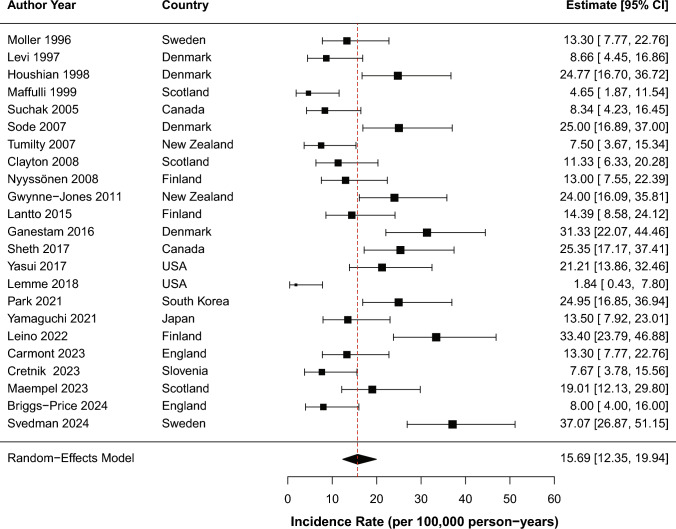
Forest plot of Achilles tendon rupture incidence rates per 100,000 person-years across 23 studies. Individual study estimates are represented by squares with horizontal lines denoting the 95% confidence intervals (CIs) calculated. The vertical dashed red line indicates the overall pooled incidence rate. The studies are ordered chronologically by publication year

The pooled overall incidence rate of ATR from 23 studies [7–13, 22, 29–34, 36, 38, 39, 41, 42, 44–47] included was estimated at 15.7 cases per 100,000 person-years (95% CI 12.4–19.9) with substantial heterogeneity (*I*^2^ = 81.6%, *Q* = 103.1, *p* < 0.0001), indicating significant variability among the included studies (Fig. 2). A sub-group analysis by country reported differences between countries. The highest IR was reported in Sweden at 22.73 (95% CI 8.33–62.02) and the lowest in the USA at 6.91 (95% CI 0.63–75.29) with substantial heterogeneity between studies in each country (Fig. S1 of the ESM). A subgroup analysis to obtain region-specific summary estimates, indicated overall differences across geographic regions (*p* < 0.0001). The IR estimates for the Americas, Asia, Europe, and Oceania were 11.18, 18.87, 16.08, and 13.96 per 100,000 person-years, respectively. Residual heterogeneity remained high after accounting for region (84.1%, *p* < 0.0001) [Fig. S2 of the ESM].

### Male:Female Ratio

**Fig. 3:**
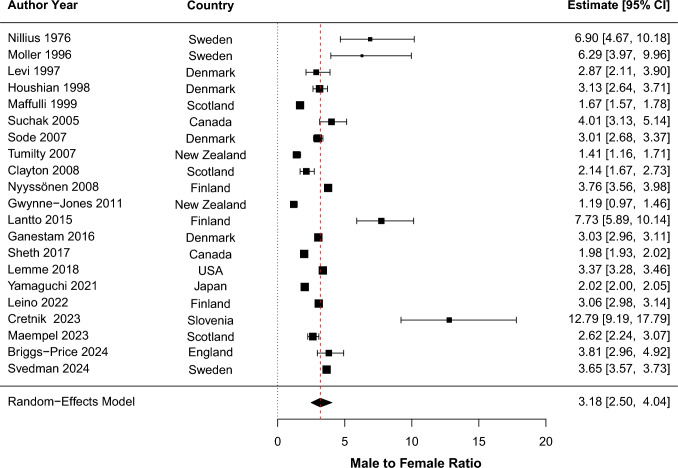
Forest plot of male-to-female ratio of Achilles tendon rupture incidence across 21 studies. Each *horizontal line* represents the 95% confidence intervals (CIs) for an individual study’s male:female ratio. The *vertical dashed red line* indicates the pooled estimate derived from a random-effects meta-analysis. Ratios greater than 1 indicate a higher incidence in male individuals compared with female individuals

The male-to-female injury ratio was available in 21 studies [7–13, 22, 28–34, 36, 39, 42, 45–47]. The random-effects meta-analysis yielded a pooled ratio of 3.18 (95% CI 2.50–4.04). Between-study heterogeneity was substantial (*τ*^2^ = 0.30; *I*^2^ = 99.86%; *Q* = 4441.9, *p* < 0.0001), suggesting considerable variability across populations and settings (Fig. 3).

### Temporal Trends of ATR

**Fig. 4:**
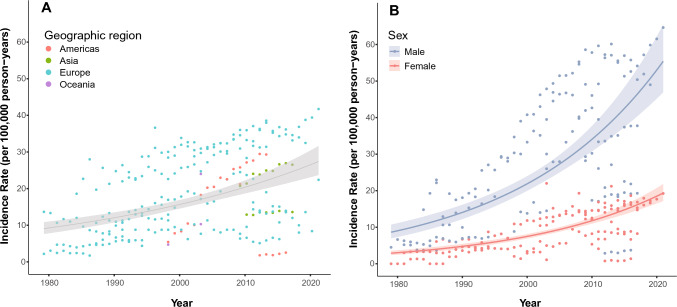
Temporal trends in the incidence of Achilles tendon rupture per 100,000 person-years across regions over time (**A**) and stratified by sex (**B**). Each point represents the incidence rate from an individual study each year. Lines indicate predicted incidence rates derived from meta-regression models for overall (gray), male (blue), and female (red) and the shaded areas represent 95% confidence intervals

To explore time-related changes, a mixed-effects meta-regression was conducted with calendar year as a moderator, estimating the trend in incidence over time and all reported incidence rates by year were included. In total, 251 timepoints were used from 23 studies [7–13, 22, 29–34, 36, 38, 39, 41, 42, 44–47]. A meta-regression showed a significant upward trend in incidence over time (*β* = 0.026, 95% CI 0.020–0.033, *p* < 0.0001), suggesting an annual increase of approximately 2.7% in ATR rates. Visual inspection of the fitted regression line and confidence bands confirmed a consistent rise in incidence from early to more recent years (Fig. 4A).

To explore the incidence rate by year as moderator and stratified by age, 284 timepoints were included from 16 studies [7, 8, 11, 13, 22, 29, 30, 33, 34, 37–39, 41, 42, 45, 46]. The pooled average log incidence rate was significantly higher in male individuals (estimate = 3.17, 95% CI 3.05–3.30) compared with females (estimate = 2.14, 95% CI 2.03–2.24), translating approximately to 23.9 and 8.5 per 100,000 person-years, respectively. A meta-regression revealed a significant increase in incidence over time for both sexes. Among male individuals, incidence increased by 4.5% annually (*β* = 0.044, 95% CI 0.036–0.052, *p* < 0.0001), while female individuals showed an annual increase of 4.6% (*β* = 0.045, 95% CI 0.038–0.052, *p* < 0.0001) [Fig. 4B].

**Fig. 5:**
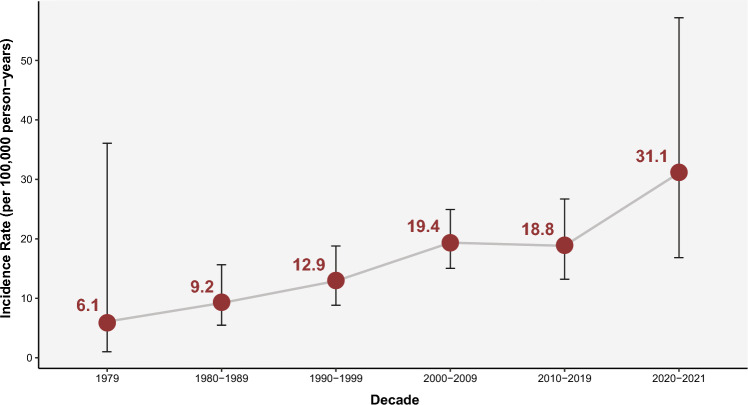
Temporal trends in the pooled incidence rate of Achilles tendon rupture by decade derived from random-effects meta-analyses, with 95% confidence intervals represented as vertical error bars. The solid line connects the point estimates across decades to illustrate the trend. Numerical values of the pooled estimates are displayed next to each point for clarity

A meta-regression was also conducted using decade as a moderator including results from 23 studies [7–13, 22, 29–34, 36, 38, 39, 41, 42, 44–47]. The model showed a significant moderator effect (QM = 12.9, *p* = 0.024), with decade accounting for 16.3% of the between-study heterogeneity. Compared with the earliest decade, incidence rates increased progressively, peaking in 2020–21 at 31.1 (95% CI 16.9–57.1) per 100,000 person-years. The estimate presents a wider CI in 1979 (two studies included [29, 34]) and in the 2020s (two studies included [11, 44]) (Fig. 5 and Fig. S3 of the ESM).

### Individual Studies Temporal Changes

The individual studies temporal changes were investigated across 17 studies (having five or more time points) [7, 8, 10, 11, 13, 22, 29–31, 34, 36, 39, 41, 42, 44–46]. All studies demonstrated an increasing trend in ATR incidence over time, except for two studies [45, 46], which showed a slight decline. Subgroup analyses by country, region, and sex consistently revealed an overall rise in ATR incidence rates (Figs. S4–S7 of the ESM).

### Incidence Rate of ATR by Age Group

**Fig. 6:**
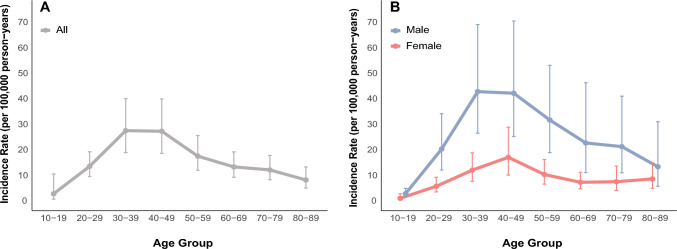
Pooled incidence rates of Achilles tendon rupture across age groups for all sexes (**A**) and stratified by sex (**B**). Blue lines and points represent data for male individuals, and orange lines and points represent data for female individuals. Each point indicates the pooled incidence rate per 100,000 person-years for a specific 10-year age group, while the vertical error bars display the corresponding 95% confidence intervals. Lines connect the age-specific estimates to highlight trends across the life span

A total of ten studies [8, 9, 28–30, 34, 37, 38, 41, 46] reported age-stratified incidence rates of ATR. Incidence increased sharply from 2.9 per 100,000 person-years in the 10–19 years of age group to a peak of 27.6 in those aged 30–39 years, with a similar high rate observed in the 40–49 years of age group (27.4). After the age of 50 years, incidence progressively declined, reaching 17.6 in the 50–59 years of age group, 13.4 in the 60–69 years of age group, and 8.3 in those aged 80–89 years. Between-study heterogeneity was substantial across most age groups (*I*^2^ > 75%), particularly among individuals aged 30–49 years (Fig. 6A). Two studies [33, 42] that were excluded from the meta-analyses reported similar trends.

A total of eight studies [8, 11, 22, 29, 32, 34, 37, 46] reported age-stratified incidence rates of ATR by sex and were included in the meta-analyses. Among male individuals, incidence increased from 2.9 per 100,000 person-years in the 10–19 years of age group to a peak of 42.9 (95% CI 26.6–69.2) in the 30–39 years of age group. Rates remained elevated in the 40–49 years of age group (42.3; 95% CI 25.3–70.6) before gradually declining with age. Female individuals exhibited a similar, though attenuated, pattern, with peak incidence observed in the 40–49 years of age group (17.2; 95% CI 10.2–29.0). Heterogeneity across age groups was substantial (*I*^2^ range from 58 to 96%), particularly in the middle-aged cohorts. Male individuals consistently showed higher incidence rates than female individuals across all age groups, based on descriptive comparisons of sex-stratified pooled estimates, with the greatest male-to-female differences observed between the ages of 30–49 years. Forest plots for each age group are presented in the Figs. S8–S15 of the ESM. The pooled incidence rates stratified by age and sex are visually summarized in Fig. 6B. Three studies [7, 13, 39] that reported age-stratified incidence rates in 20-year intervals were excluded from the meta-analyses but showed similar trends.

### Incidence Rate of ATR in Sports

**Fig. 7:**
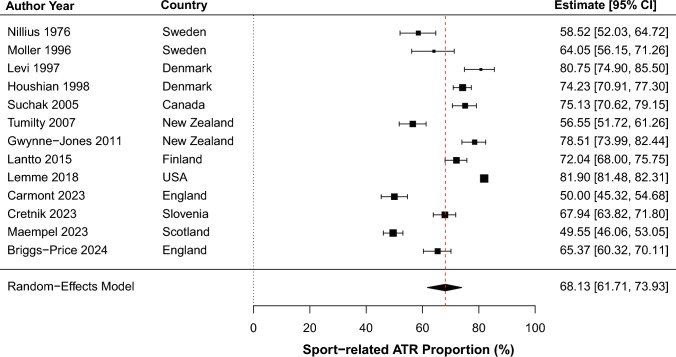
Forest plots showing the pooled proportion of sport-related Achilles tendon rupture (ATRs) calculated using a random-effects meta-analysis. Each square represents an individual study estimate, with horizontal lines indicating the 95% confidence intervals (CIs). The red vertical dashed line indicates the pooled estimate. Results are presented as percentages on the *x-axis*, back-transformed from the logit scale

Thirteen studies [9, 12, 22, 28–30, 33, 34, 39, 44–47] reported the proportion of ATRs attributed to sports participation. The pooled estimate indicated that 68.1% (95% CI 61.7–73.9) of ruptures were sport related. However, heterogeneity across studies was substantial (*I*^2^ = 97.7%) [Fig. 7].

### Incidence Rates by Age Group and Sport Participation

In total, three studies [28, 30, 34] reported incidence rates stratified by age group and injury mechanism (sport vs non-sport). Among individuals participating in sport, the highest pooled incidence rates were observed in the 30–39 and 40–49 years of age groups, with estimates of 28.11 (95% CI 6.39–123.56) and 25.33 (95% CI 5.58–114.89) per 100,000 person-years, respectively. In contrast, incidence rates among non-sport participants peaked later, in the 50–59 and 60–69 years of age groups, at 11.62 (95% CI 5.74–23.53) and 11.40 (95% CI 4.25–30.63) per 100,000 person-years, respectively (Fig. S16 of the ESM).

### Surgical Temporal Trend

**Fig. 8:**
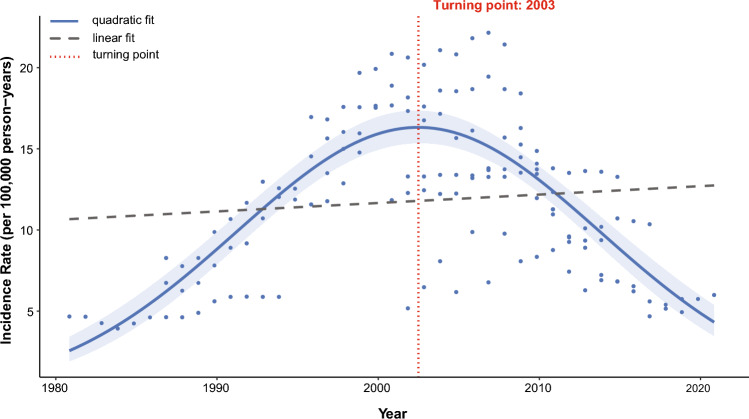
Quadratic meta-regression of log-transformed surgical incidence rates across 162 timepoints. The *blue line* shows the estimated trend with 95% confidence intervals (shaded). The dashed gray line represents a basic linear trend for comparison. The red dotted line marks the year of peak surgical incidence (2003). Each dot represents a study estimate (rate per 100,000 person-years). The quadratic model explained all between-study heterogeneity (*R*^2^ = 100%) and fit significantly better than the linear model (*p* < 0.0001)

In total, ten studies [7, 8, 10, 11, 13, 35, 40–43] reported annual incidence rates of surgical repair following ATRs. The quadratic meta-regression analysis revealed a significant non-linear trend over time. Based on 145 data points, the model demonstrated excellent fit with no residual heterogeneity (*τ*^2^ = 0, *I*^2^ = 0%), and accounted for 100% of the observed variance (*R*^2^ = 100%). The regression identified a statistically significant turning point in 2002.7, indicating an increasing trend in surgical incidence up to that year, followed by a decline thereafter. All regression coefficients were statistically significant (*p* < 0.0001), including the negative coefficient for the year^2^ term, confirming a concave (downward-sloping) temporal trend (Fig. 8).

### Seasonality

Eight studies investigated the seasonality of ATR incidence rates [7, 8, 10, 22, 37, 41, 44, 46]. Findings across studies were mixed, with no clear consensus on the influence of season. However, four studies observed a trend toward higher ATR incidence during the summer months [37, 41, 44, 46, 48] (Table 1).

## Discussion

This meta-analysis synthesizes data from 28 population-based studies conducted between 1950 and 2022, covering over 630 million individuals and documenting nearly 563,000 ATRs. The goal was to estimate the global incidence of ATR, assess temporal trends, examine sex-based and age-specific differences, and evaluate geographic and treatment-related variations. The key findings reveal an increasing incidence over time with an incidence rate of 6.1 in 1979 and an average incidence rate the current decade of 31.1 per 100,000 persons. There is a higher incidence in male individuals (almost three-fold compared with male individuals), aged 30–50 years, and those who are actively engaged in sports. There is a temporal trend for almost a 5% annual increase in incidence rates in both male and female individuals. There is also a declining surgical trend post-2003. To the best of our knowledge, this is the first study to provide synthesis and temporal trends of incidence estimates for ATR.

### Temporal Trends and Sport-Related Etiology in ATR

The results of the meta-analysis highlighted a consistent long-term increase in the incidence of ATRs over the past six decades. The analysis showed a statistically significant average annual increase of 2.7% in incidence rates. When incidence data were grouped by decade, the trend was particularly notable: pooled incidence rate rose from 6 per 100,000 person-years in 1979, to 9 in the 1980s, 13 in the 1990s, and 19 in both the 2000s and 2010s, reflecting a near five-fold increase in reported rates from 1979 to the early 2020s.

Interestingly, this upward trajectory appeared to plateau during the 2010s, remaining at approximately 19 cases per 100,000 person-years. Several large epidemiological studies included in the analysis reported a slowed increase [11, 41, 44], no change [7, 13], or even a slight decline [45, 46] during this period. This temporary stabilization may be partially explained by the statistical pooling methods or social and lifestyle changes, but such interpretations remain speculative and require further validation.

Following this plateau, the most recent data indicate a renewed increase in ATR incidence, with pooled estimates rising to approximately 31 per 100,000 person-years, raising concern for a new wave of tendon injuries in the general population. However, this finding is currently supported by only two large studies [11, 44] and should be interpreted with caution. One plausible explanation for this sharp increase the last decade is the rapid global rise in popularity of racquet sports, such as padel and pickleball. In the USA, pickleball is the fastest-growing sport, with participation nearly doubling from 4.8 million in 2022 to 8.9 million in 2023 [49, 50]. Between 2015 and 2023, the incidence of pickleball-related foot and ankle injuries, increased significantly, particularly among older male patients [51]. In a recent study of 166 outpatient cases involving pickleball and paddleball injuries, 12% sustained an ATR, with 88% of those managed surgically [52].

### Sport- and Age-Related Etiology

The results of this meta-analysis confirm the predominant role of sports participation in the etiology of ATR, with an estimated 68% of ruptures occurring during sport-related activities across the included studies. Age-stratified analysis of ATR reveals a distinct peak in incidence among middle-aged adults, particularly men aged 30–49 years. The highest rate was observed in the 30–39 years of age group (42.9 per 100,000), with similarly elevated rates in the 40–49 years of age group. Women showed a similar pattern but at lower rates, peaking at 17.2 per 100,000 in the 40–49 years of age range. These peaks are primarily linked to sport-related injuries, whereas non-sport-related ATRs tend to occur later in life, with the highest incidence in the 60–69 years of age group. Pooled incidence rates of ATR, stratified by mechanism of injury, showed that for sport-related injuries the peak age was 30–39 years, whereas for non-sport-related injuries the peak age was 60–69 years. However, only three studies [28, 30, 34] were included in this subgroup analysis, so the results should be interpreted with caution.

The reviewed literature revealed a general lack of a standardized definition for sport-related injury. Furthermore, the reporting of injury mechanisms was typically limited to a simple “sport” or “non-sport” binary classification, and detailed contextual information regarding the specific activity at the time of injury was infrequently provided. Importantly, despite the high proportion of sport-related ATRs, most studies provided limited detail regarding the level of athletic participation, making it difficult to distinguish between recreational and competitive athletes. The existing literature suggests that only 4–18% of ATRs occur in competitive athletes [45, 53]. Despite this relatively small proportion, the impact of ATR in elite athletes is substantial. Among recreational athletes, about 80% return to play within 6 months [54, 55]. Among professional athletes, return-to-play rates range from 61 to 100%, with approximately 30% unable to return within 2 years, an average return time of 11 months, and some ending their careers [56, 57]. Moreover, elite athletes often struggle to regain pre-injury performance, particularly in high-demand sports such as basketball [57].

The literature reports a statistically significant rise in mean age of ATR patients over time [21]. This age-related shift in ATR is likely due to both increased participation in physical activity and age-related tendon degeneration. As people age, tendon stiffness decreases, leading to increased strain with functional activities [58]. This cumulative tissue fatigue may result in tissue failure. This shift is seen globally, with countries such as Sweden reporting a five-fold higher ATR incidence in middle-aged individuals today compared with their younger years [11]. The phenomenon reflects the growing popularity of active aging, where people in their 40s, 50s, and beyond remain highly active, engaging in sports and recreational activities that can stress aging musculoskeletal structures. However, not all regions mirror this trend; in Japan, for example, ATR rates remain relatively constant across age groups, potentially owing to lower engagement in high-impact sports among older adults [13].

### Geographic Variability

Although temporal trends in ATR incidence appear broadly consistent across geographic regions, absolute incidence rates vary considerably by country. These differences likely reflect variations in physical activity levels, sport participation, cultural socioeconomic factors, and the quality of injury surveillance systems. However, these findings should be interpreted with caution, as the wide CIs observed in country-specific subgroup analyses indicate considerable uncertainty—largely attributable to differences in study periods and the influence of time on incidence estimates.

Among countries, Scandinavian nations, particularly Sweden and Denmark, reported the highest national incidence (22.7 and 21.5 per 100,000, respectively). This elevated rate likely stems from a combination of high recreational sport participation and the presence of a comprehensive national injury registry, which facilitates more complete and accurate case reporting [59, 60]. Conversely, the USA (and many other countries) recorded the lowest incidence (6.9 per 100,000) plausibly due to (i) the absence of a mandatory, comprehensive, national injury registry and reliance on claims/hospital databases that miss many uninsured, self-pay, and outpatient cases [61] and (ii) lower population-level physical activity, which may reduce exposure to sport-related injury but carries a far greater overall public health burden [62].

Interestingly, ATR appears to be more common in socioeconomically advantaged populations [46], a pattern that contrasts with most other health conditions. One possible explanation is that individuals from higher income backgrounds are more likely to participate in structured exercise and recreational sports, increasing their exposure to tendon-loading activities and associated injury risk. Additionally, they are more likely to engage in individual sports, possibly owing to better access to facilities and the financial means to support such activities [63–65]. The influence of healthcare access on ATR incidence is likely present [66, 67], but probably limited, given the injury’s acute and painful nature, which typically prompts medical attention regardless of healthcare system. Finally, the types of popular sports within each country also contribute to national variations in ATR incidence. For instance, badminton and soccer are common causes in Scandinavia [7, 30, 34], basketball in the USA [39], and netball and squash in New Zealand [12, 33].

### Sex-Based Differences

A consistent and robust finding of this meta-analysis was the male predominance in ATR rates. The pooled male-to-female ratio was 3.18, indicating that men are over three times more likely to experience ATRs than women. This aligns with previous literature attributing higher rupture rates in male individuals because of greater participation in high-impact sports, higher tendon-loading activities, and potentially sex-specific biomechanical and hormonal differences [34, 45]. However, the male-to-female ratio varied substantially across countries (range from 1.2 to 12.8), likely reflecting sociocultural differences in sport participation and physical activity patterns. For instance, the sex ratio was approximately 1:1 in New Zealand, 3:1 in Denmark, the USA, and Canada, 2:1 in Scotland and Japan, and as high as 13:1 in Slovenia.

Importantly, despite the overall male predominance, ATR incidence increased over time in both sexes. The annual increase was similar in male individuals (4.5% per year) and female individuals (4.6%). Age/exposure shaped the sex-specific injury profile: men aged 30–49 years more often had ATRs during basketball or soccer, whereas women aged over 50 years more often had non-sports-related ruptures and, when sport related, during handball or volleyball [20, 30, 68].

There are well-documented sex-based differences in tendon morphology, physiology, and mechanical properties. Biomechanical studies have shown that men have longer, thicker, and stiffer Achilles tendons, with lower compliance and greater daily loading [69]. In contrast, female individuals demonstrate lower tendon stiffness, a smaller cross-sectional area, and reduced adaptability to mechanical loading [70]. However, the current evidence does not support a direct or causal relationship between these tendon characteristics and tendon injury risk differences between sexes [71]. In fact, a recent study suggests these disparities may diminish when adjusting for physical activity and body weight, highlighting the importance of lifestyle factors [72].

### Shifting Trends in Surgical Treatment

One of the most significant findings of this meta-analysis is the evolving trend in the surgical management of ATR, marked by a clear turning point in 2003. The quadratic model applied to 145 timepoints showed surgical repair rates rising steadily until 2003, after which a notable decline began. The model showed excellent fit (*τ*^2^ = 0, *I*^2^ = 0%), suggesting that this shift is robust and globally reflective of changes in clinical practice driven by evidence-based medicine.

The decline in surgical treatment rates aligns with trends in countries such as those in northern Europe and Canada [11, 35, 36, 42]. The preference for conservative treatment is based on evidence showing similar long-term functional outcomes with fewer adverse events, though it may carry a slightly higher re-rupture risk [5, 6, 15]. However, this does not apply uniformly. Recent data from Japan and South Korea show increased surgical treatment rates, likely influenced by healthcare differences, such as private care dominance and poor outpatient support for non-surgical protocols [13, 41, 43].

Still, there is no clear consensus on how to optimally select patients for surgical versus conservative care. Historically, surgery was the dominant treatment, but over time, conservative management gained acceptance, especially among older individuals. In the USA for example, patients aged over 85 years are much more likely to be treated nonoperatively [73]. Furthermore, sex-based differences in tendon healing have emerged: women report greater deficits in heel-rise height and more symptoms post-surgery than men, but these differences are not evident after conservative treatment [74].

Young patients might undergo surgery more often compared with older individuals, likely owing to their higher functional demands and fewer comorbidities. Among elite athletes, surgical repair remains the dominant choice [68, 75]. These individuals often face intense pressure from teams, sponsors, and media to return to competition as quickly as possible, making rapid functional recovery a top priority. In this context, surgery is often selected despite the risks, as it may offer a more predictable timeline for rehabilitation and return to sport. Studies of elite athletes undergoing percutaneous repair and early mobilization protocols reported return to sport as early as 19 weeks post-surgery [48]. Despite this, return-to-performance outcomes remain suboptimal, and studies have noted long-term deficits in strength and muscle mass even among elite patients [48].

From a cost-effectiveness perspective, surgery is generally more expensive but may be justified depending on healthcare system thresholds and patient goals [76, 77]. Healthcare resources can be saved if ATRs are detected at an early stage [78], but still there are reports for 20% of missed diagnosis [79].

### Strengths and Limitations

This systematic review and meta-analysis is the most comprehensive synthesis of ATR incidence to date, incorporating data from four continents over a span of six decades. Its primary strengths include the use of robust statistical techniques—random-effects models, log transformations, and extensive moderator analyses—to account for heterogeneity and enhance generalizability. The stratification of data by sex, age group, region, and decade allowed for a detailed examination of temporal and geographic trends. Importantly, the inclusion of population-based studies, particularly those using nationwide registries, minimized selection bias and improved the reliability of incidence estimates compared with studies based on single-center cohorts.

Despite these strengths, several limitations must be acknowledged. The majority of studies were conducted in high-income countries, especially in Scandinavia, where strong injury surveillance systems exist. This geographic imbalance limits the applicability of findings to low- and middle-income countries, where data are scarce. No studies were identified from Africa or Latin America, and only a few were available from Asia.

Considerable between-study heterogeneity was observed (I^2^ often exceeding 80%), reflecting differences in study design, case definitions, healthcare access, and coding practices. Additional sources of bias include reliance on administrative data, which lack clinical detail, and inconsistent inclusion criteria across studies (e.g., insured, aged older than 18 years, index injury). Furthermore, misdiagnosis or misclassification may occur, especially in partial or chronic ruptures, potentially leading to under-reporting and underestimation of ATR incidence. Subnational variations, socioeconomic disparities, and racial or ethnic factors were not addressed because of a lack of data to synthesize the results. We did not account for other factors such as medication use (e.g., corticosteroids, quinolones, bisphosphonates), obesity, chronic tendon inflammation, diabetes mellitus, genetic predisposition, or systemic conditions such as renal failure and hyperparathyroidism. Each of these has been suggested as a potential risk factor for ATR, though current evidence remains limited or of low certainty [80].

The literature does not consistently report the activity level of the injured population (e.g., elite vs recreational athletes, or whether participants were regularly active or only occasionally involved in sports). Future studies should aim to clarify this aspect, as this is an important source of variability. In addition, classifications of sport versus non-sport related injuries were not consistent across studies. We accepted each study’s author-defined categories; while the high-level dichotomy is similar, differences in operational definition likely contributed to the observed heterogeneity.

### Clinical Implications and Future Research

The global incidence of ATR is steadily rising, especially among middle-aged men who engage in recreational sports. Health systems must be prepared for the increasing burden of ATR, which often requires specialized and extended rehabilitation. Early diagnosis remains essential, particularly in less active individuals who may not recognize the symptoms of ATR. As conservative management becomes more common, there is an urgent demand for evidence-based rehabilitation protocols that ensure optimal healing, functional recovery, and reduced reinjury risk.

The popularity of high-risk sports such as ball and racquet games, combined with aging populations, suggests that ATR incidence will continue to rise. This underscores the need for coordinated efforts among governments, sports organizations, and healthcare providers to develop and scale prevention and rehabilitation frameworks, based on robust evidence, suited to real-world settings and adjusted for each sport. Aligned with van Mechelen’s “sequence of prevention,” injury surveillance must first establish ATR incidence and severity, followed by identifying etiology and mechanisms. Prevention measures can then be designed and evaluated effectively, with tailored surveillance systems needed to address sport-specific injury risks.

Future research should focus on addressing critical knowledge gaps in ATR epidemiology and management. Most urgently, prospective population-based studies from under-represented regions, particularly Africa, Asia, and Latin America are needed to provide a more accurate global picture. These studies should consider country-specific healthcare systems, activity patterns, and cultural contexts that may influence incidence and treatment. Further investigation into factors contributing to heterogeneity, including socioeconomic status, race, and healthcare accessibility is essential. Future research should focus on improving study designs and measurement approaches to better isolate the contributions of biological, behavioral, and mechanical factors in sex-specific injury risk. In addition, age- and sex-specific analyses of recurrent rupture, functional recovery, and long-term quality of life would enable more tailored treatment strategies. As the debate between operative and non-operative treatment continues, large-scale registries with patient-reported outcomes and stratified risk profiles (e.g., recreational vs professional athletes) could help refine current treatment algorithms and long-term tracking of outcomes and reinjury rates across treatment pathways.

## Conclusions

This meta-analysis reports a rising global increase in ATR incidence over the past 60 years, with an average annual rise of 2.7% and a peak pooled incidence of 31.1 per 100,000 person-years in the 2020s. Pronounced sex and age disparities were observed: men are over three times more likely to experience ATR than women (male-to-female ratio 3.18), with peak incidence occurring in men aged 30–49 years and women aged 40–49 years. Approximately 68% of ATRs are linked to sports participation, particularly ball and racquet sports. Surgical treatment rates peaked around 2003 and have since declined, reflecting evolving clinical practice.

## Supplementary Information

Below is the link to the electronic supplementary material.

Supplementary file1 (PDF 1703 KB)

## Declarations

### Availability of Data and Material

All information supporting the findings of this study are available within the paper and its supplementary information.
