# Supplementary material for: Incidence, Temporal Trends, and Surgical Shift of Achilles Tendon Rupture: A Systematic Review and Meta-analysis
Source: Sports Med. 2026 Apr 3;56(6):1467–87. doi: 10.1007/s40279-026-02397-5 (PMC13260312; doi:10.1007/s40279-026-02397-5)
Supplement: Supplementary file 1 — Supplementary file1 (PDF 1703 KB) [file 40279_2026_2397_MOESM1_ESM.pdf]

Incidence, temporal trends, and surgical shift of Achilles Tendon rupture: A systematic review and meta-analysis

Supplementary file

Sports Medicine

Authors:

Roula Kotsifaki[1,2], Peter Malliaras[3], Christopher Byron[3], Joao Marques[1], Vasileios Korakakis[4]

Affiliations:

1. Rehabilitation Department, Aspetar, Orthopaedic and Sports Medicine Hospital, Doha, Qatar
2. Oslo Sports Trauma Research Center, Department of Sports Medicine, Norwegian School of Sport Sciences, Oslo, Norway
3. Physiotherapy Department, School of Primary and Allied Health Care, Monash University, Melbourne, Australia
4. Department of Health Sciences, School of Life Sciences and Health Sciences, PhD in Physiotherapy Program, University of Nicosia, Cyprus

Corresponding author's contact details:

Roula Kotsifaki: Sports City Street, P.O. Box 29222, Doha, Qatar, Argyro.Kotsifaki@aspetar.com

Table S1. Search strategy

Pubmed

S1	(((achilles) OR (achilles tendon[MeSH Terms])) OR (achill*[Title/Abstract])) OR (tendoachill*[Title/Abstract])
S2	(((((rupture[MeSH Terms]) OR (tendon injuries[MeSH Terms])) OR (ruptur*[Title/Abstract])) OR (injur*[Title/Abstract])) OR (lesion*[Title/Abstract])) OR (tear*[Title/Abstract])
S3	((((((((((((epidemiology[MeSH Terms]) OR epidemiolog*[Title/Abstract]) OR (survey[Title/Abstract])) OR (pattern*[Title/Abstract])) OR (incidence*[Title/Abstract])) OR (prevalence*[Title/Abstract])) OR (incidence[MeSH Terms])) OR (prevalence[MeSH Terms])) OR (exposure[Title/Abstract])) OR (audit*[Title/Abstract])) OR (rate[Title/Abstract])) OR (burden[Title/Abstract])) OR (frequenc*[Title/Abstract])) OR Surveillance [Title/Abstract])
S4	S1 AND S2 AND S3 – Filter: only humans

EMBASE

S1	(((exp achilles tendon/ or achilles.mp)) OR (achill*.ti,ab)) OR (tendoachill*.ti,ab)
S2	((((((exp achilles tendon rupture/ or rupture.mp. Or exp rupture/ or exp tendon rupture/)) OR (tendon injuries.mp. or exp tendon injury/)) OR (ruptur*.ti,ab)) OR (injur*.ti,ab)) OR (lesion*.ti,ab)) OR (tear*.ti,ab)
S3	(((((((((((((exp epidemiology/ or epidemiology.mp)) OR (epidemiology*.ti,ab)) OR (survey.ti,ab)) OR (pattern*.ti,ab)) OR (incidence*.ti,ab)) OR (prevalence*.ti,ab)) OR (incidence.ti,ab)) OR (prevalence*.ti,ab)) OR (exposure, ti.ab)) OR (audit*.ti,ab)) OR (rate.ti,ab)) OR (burden.ti,ab)) OR (frequenc*.ti,ab)) OR (surveillance.ti,ab)
S4	S1 AND S2 AND S3 – Filter: only humans

Web of science

S1	TS=(achilles tendon OR achill* OR tendoachill*)
S2	TS=(ruptur* OR injur* OR lesion* OR tear*)
S3	TS=(epidemiolog* OR incidence OR prevalence OR pattern* OR survey OR exposure OR audit OR rate OR burden OR frequenc* OR surveillance)
S4	S1 AND S2 AND S3 (no filter available)

CINAHL

S1 achilles OR tendoachill*

S2 ruptur* OR injur* or lesion* or tear*

S3 epidemiolog* OR incidence OR prevalence OR survey OR rate OR burden OR exposure OR audit* OR frequenc* OR surveillance

S4 S1 AND S2 AND S3 – Filter: only humans

Global Index Medicus

S1 achilles OR tendoachill* (TITLE, ABSTRACT, SUBJECT)

S2 ruptur* OR injur* or lesion* or tear* (TITLE, ABSTRACT, SUBJECT)

S3 epidemiolog* OR incidence OR prevalence OR survey OR rate OR burden OR exposure OR audit* OR frequenc* OR surveillance (TITLE, ABSTRACT, SUBJECT)

S4 S1 AND S2 AND S3 (no filter available)

Google scholar

achilles AND rupture AND incidence

Table S2. Checklist for Prevalence Studies developed by the Joanna Briggs Institute

Author & year	Q1 (sample frame)	Q2 (recruitment)	Q3 (sample size)	Q4 (Subjects & setting)	Q5 (coverage)	Q6 (identification of the condition)	Q7 (measurement)	Q8 (statistics)	Q9 (response rate)
**Nillius 1976 [1]**	YES	YES	YES	YES	YES	YES	YES	UNCLEAR	NA
**Moller 1996 [2]**	YES	YES	YES	YES	YES	YES	YES	YES	NA
**Levi 1997 [3]**	YES	YES	YES	YES	YES	YES	YES	YES	NA
**Houshian 1998 [4]**	YES	YES	YES	YES	YES	YES	YES	YES	NA
**Maffulli 1999 [5]**	YES	YES	YES	YES	YES	YES	YES	YES	NA
**Suchak 2005 [6]**	YES	YES	YES	YES	YES	YES	YES	YES	NA
**Sode 2007 [7]**	YES	YES	YES	YES	YES	YES	YES	YES	NA
**Tumilty 2007 [8]**	YES	YES	YES	YES	NO	YES	YES	NO	NA
**Clayton 2008 [9]**	YES	YES	YES	YES	YES	YES	YES	UNCLEAR	NA
**Nyyssönen 2008 [10]**	YES	YES	YES	YES	YES	YES	YES	YES	NA
**Gwynne-Jones 2011 [11]**	NO	YES	YES	YES	YES	YES	YES	YES	NA
**Lantto 2015 [12]**	YES	YES	YES	YES	YES	YES	YES	YES	NA
**Mattila 2015 [13]**	YES	YES	YES	YES	YES	YES	YES	YES	NA
**Ganestam 2016 [14]**	YES	YES	YES	YES	YES	YES	YES	YES	NA
**Sheth-a 2017 [15]**	YES	YES	YES	YES	YES	YES	YES	YES	NA
**Sheth-b 2017 [16]**	YES	YES	YES	YES	YES	YES	YES	YES	NA
**Yasui 2017 [17]**	NO	YES	YES	YES	YES	YES	YES	UNCLEAR	NA
**Lemme 2018 [18]**	YES	YES	YES	YES	YES	YES	YES	YES	NA
**Longo 2020 [19]**	YES	YES	YES	YES	YES	YES	YES	YES	NA
**Park 2021 [20]**	YES	YES	YES	YES	YES	YES	YES	YES	NA
**Yamaguchi 2021 [21]**	YES	YES	YES	YES	YES	YES	YES	YES	NA
**Leino 2022 [22]**	YES	YES	YES	YES	YES	YES	YES	YES	NA
**Park 2022 [23]**	YES	YES	YES	YES	YES	YES	YES	YES	NA
**Carmont 2023 [24]**	YES	NO	YES	YES	YES	YES	YES	UNCLEAR	NA
**Cretnik 2023 [25]**	UNCLEAR	YES	YES	YES	YES	YES	YES	YES	NA
**Maempel 2023 [26]**	YES	YES	YES	YES	YES	YES	YES	YES	NA
**Briggs-Price 2024 [27]**	NO	YES	YES	UNCLEAR	YES	YES	YES	UNCLEAR	NA
**Svedman 2024 [28]**	YES	YES	YES	YES	YES	YES	YES	YES	NA

Overall IR by country

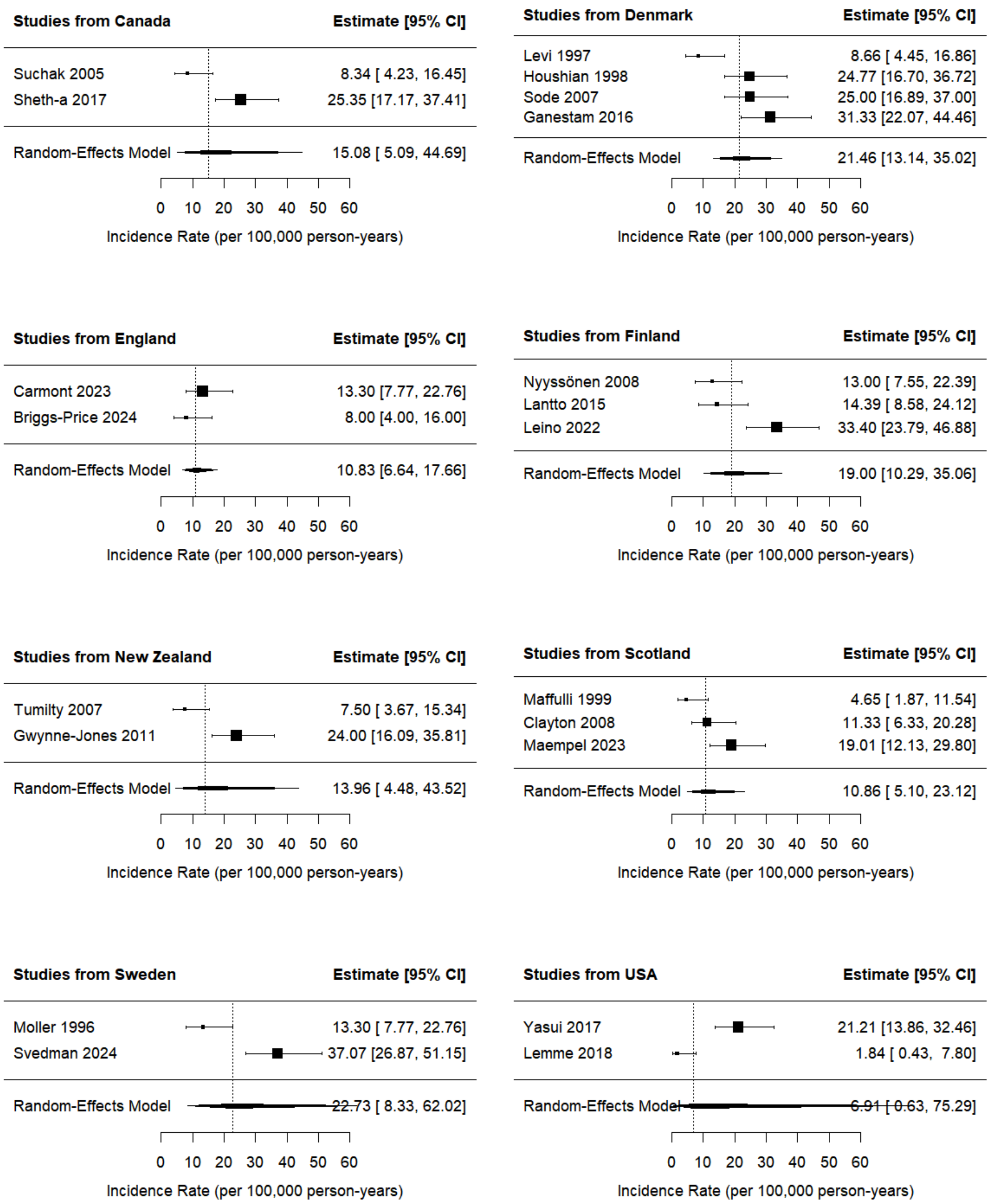

Fig. S1 Forest plots of Achilles tendon rupture incidence rates (per 100,000 person-years) by country. Each study's effect estimate is shown as a square with a horizontal line representing its 95% confidence interval for the incidence rate per 100,000 person-years. The diamond indicates the overall pooled estimate from the meta-analysis, with the x-axis displaying the back-transformed incidence rates.

Overall IR by continent

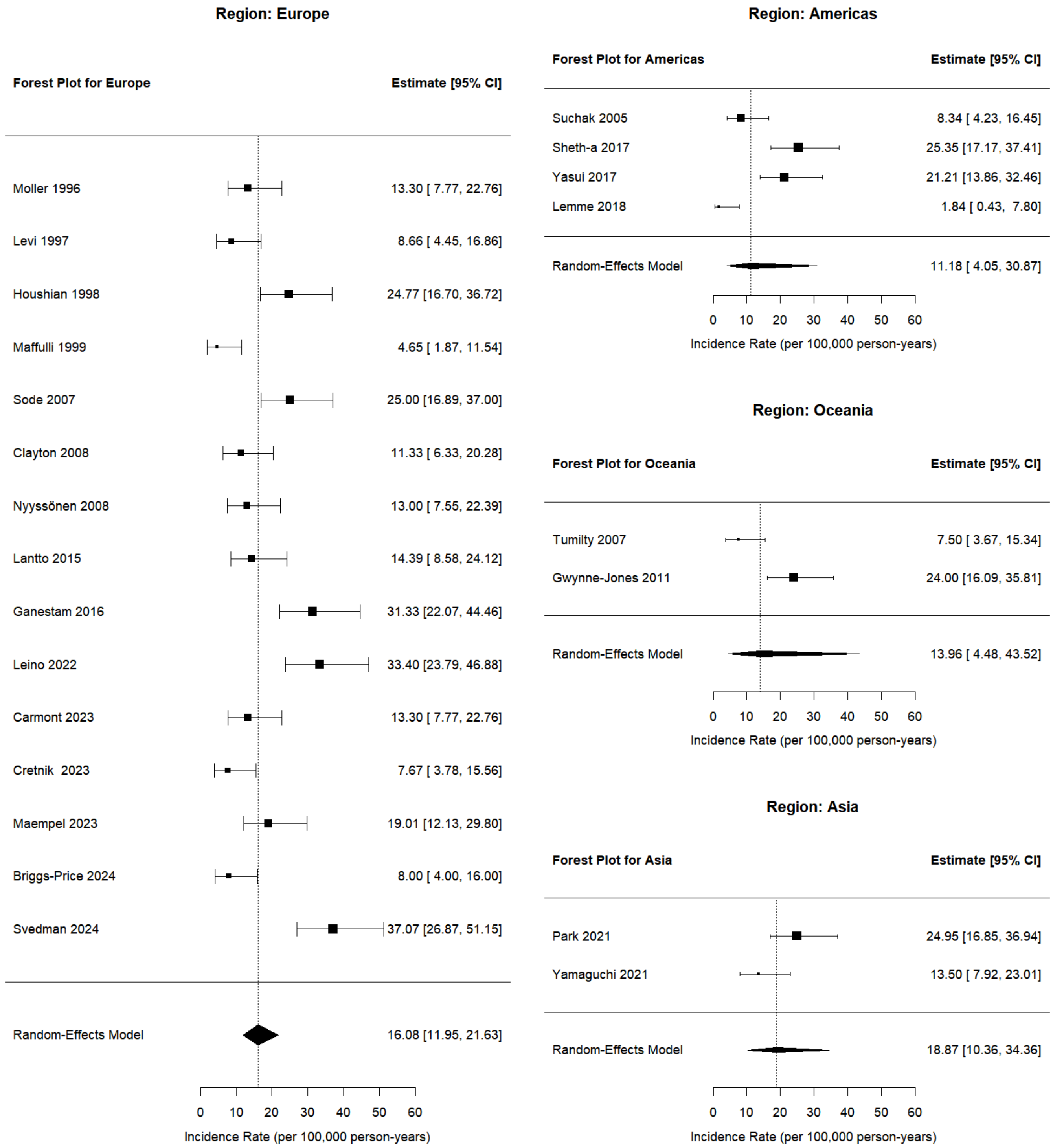

Fig. S2 Forest plots of Achilles tendon rupture incidence rates (per 100,000 person-years) by region. Each subplot presents a random-effects meta-analysis of studies within a specific region, with studies ordered by publication year. The horizontal lines represent 95% confidence intervals, and the vertical dotted line represents the regional pooled incidence estimate for reference.

Incidence rates by decade

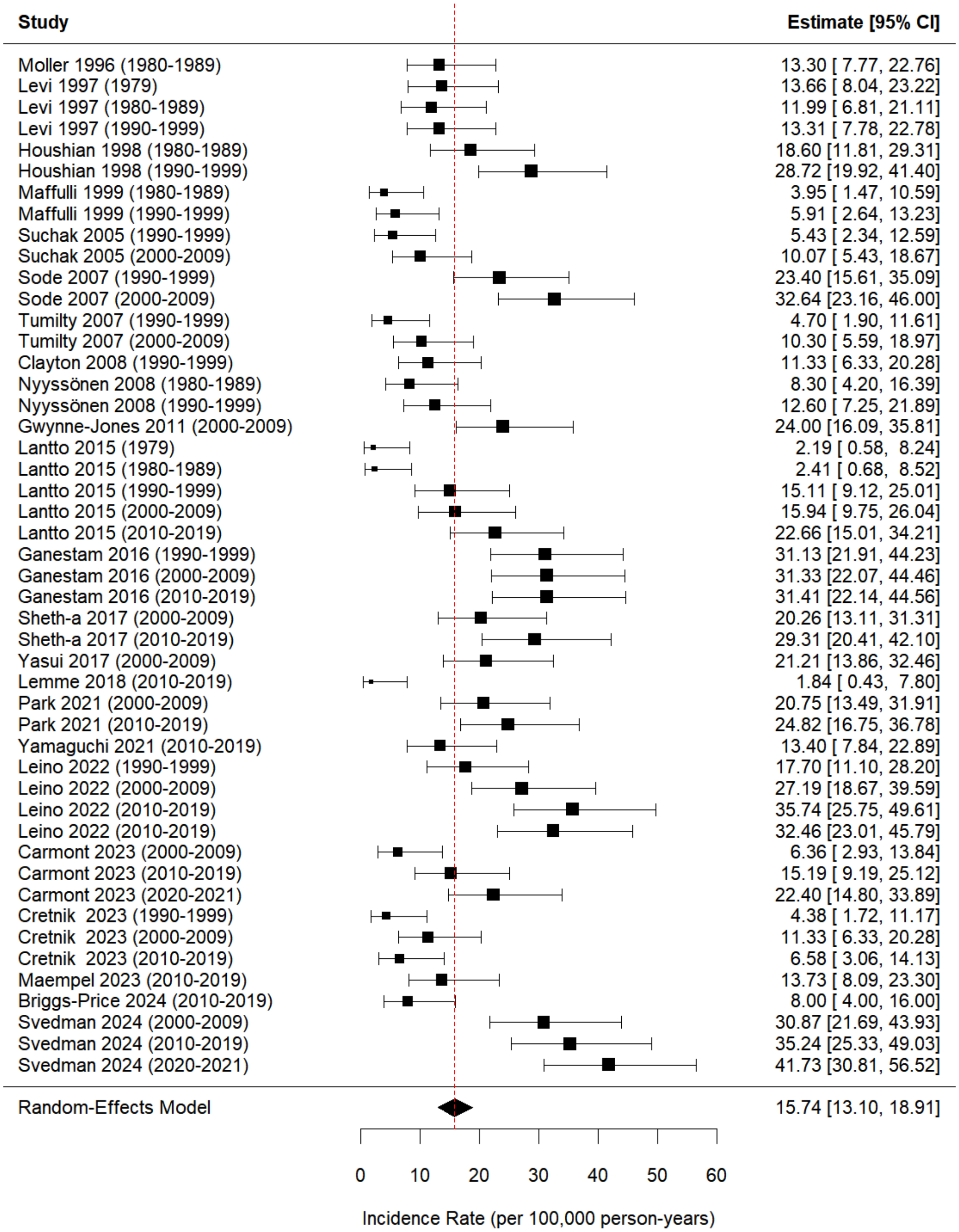

Fig. S3 Forest plot of Achilles tendon rupture incidence rates (per 100,000 person-years) by decade. Each horizontal line represents a study-specific incidence rate with its 95% confidence interval. For studies spanning multiple decades, the data point closest to the mid-decade was used. Studies are ordered chronologically by publication year The vertical line indicates the pooled estimate.

Individual studies temporal changes

Individual studies temporal changes - All

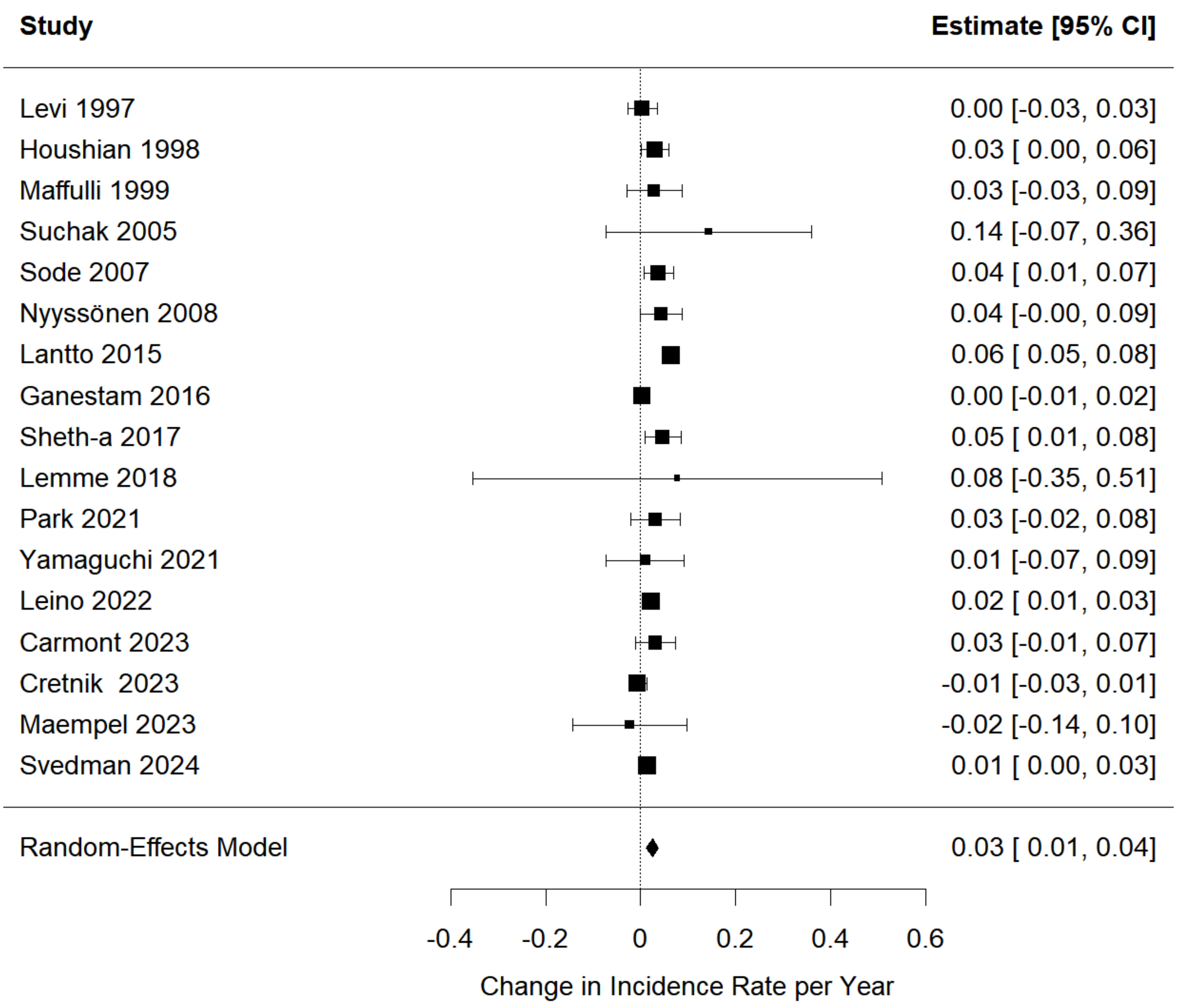

Fig. S4 Forest plot of temporal changes within each study of the incidence of Achilles tendon rupture. Each study's temporal trend was estimated using Poisson regression. Only studies which report at least five points were included. The overall pooled estimate from a random-effects meta-analysis is shown as a diamond. Error bars represent 95% confidence intervals.

Individual studies temporal changes by country

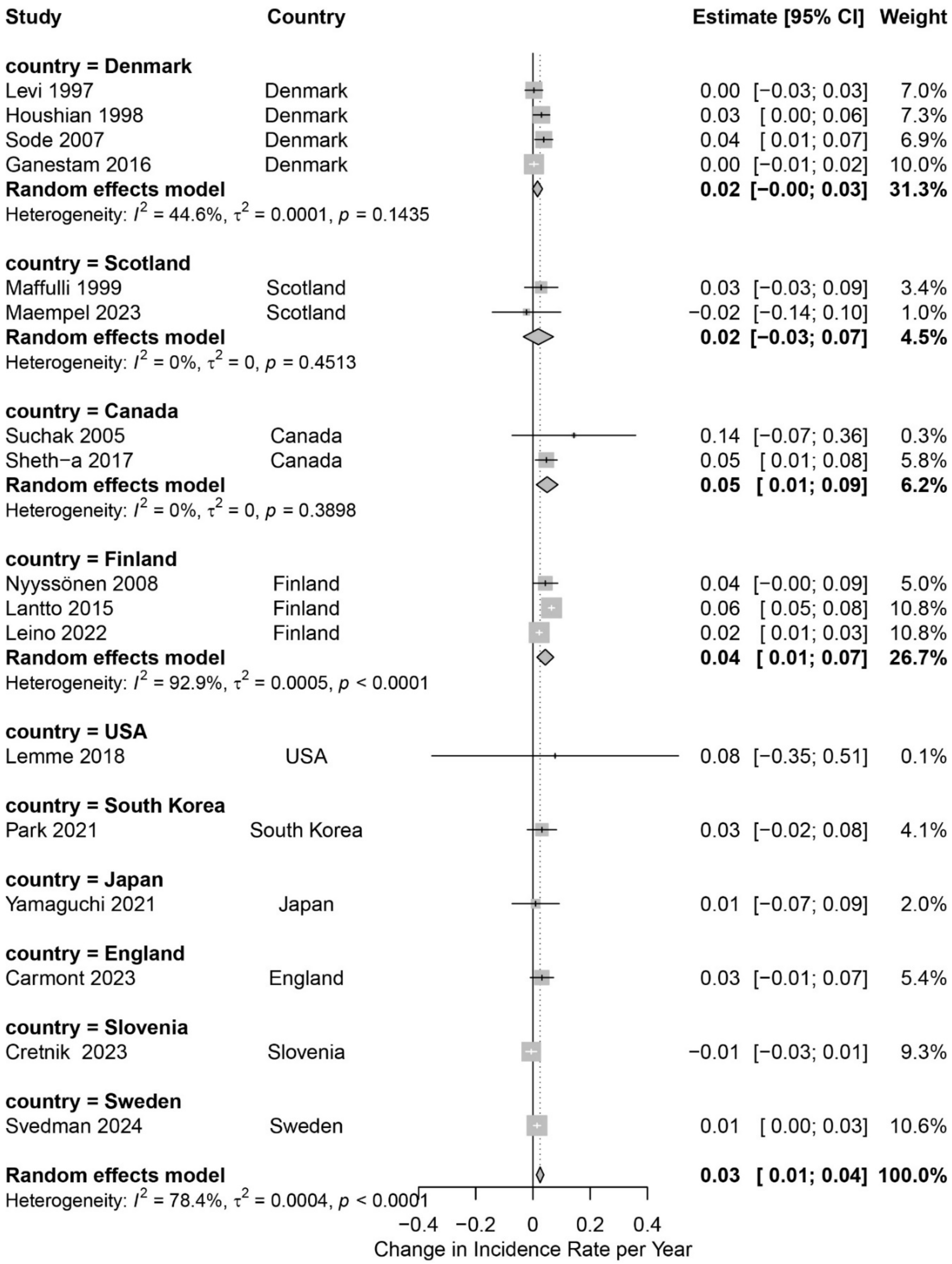

Fig. S5. Forest plot of temporal trends in Achilles tendon rupture incidence, stratified by country. Within-study changes over time are displayed with 95% confidence intervals, and where applicable, pooled estimates are calculated for each country. Diamond markers denote the country-specific pooled incidence trends.

Individual studies temporal changes by continent

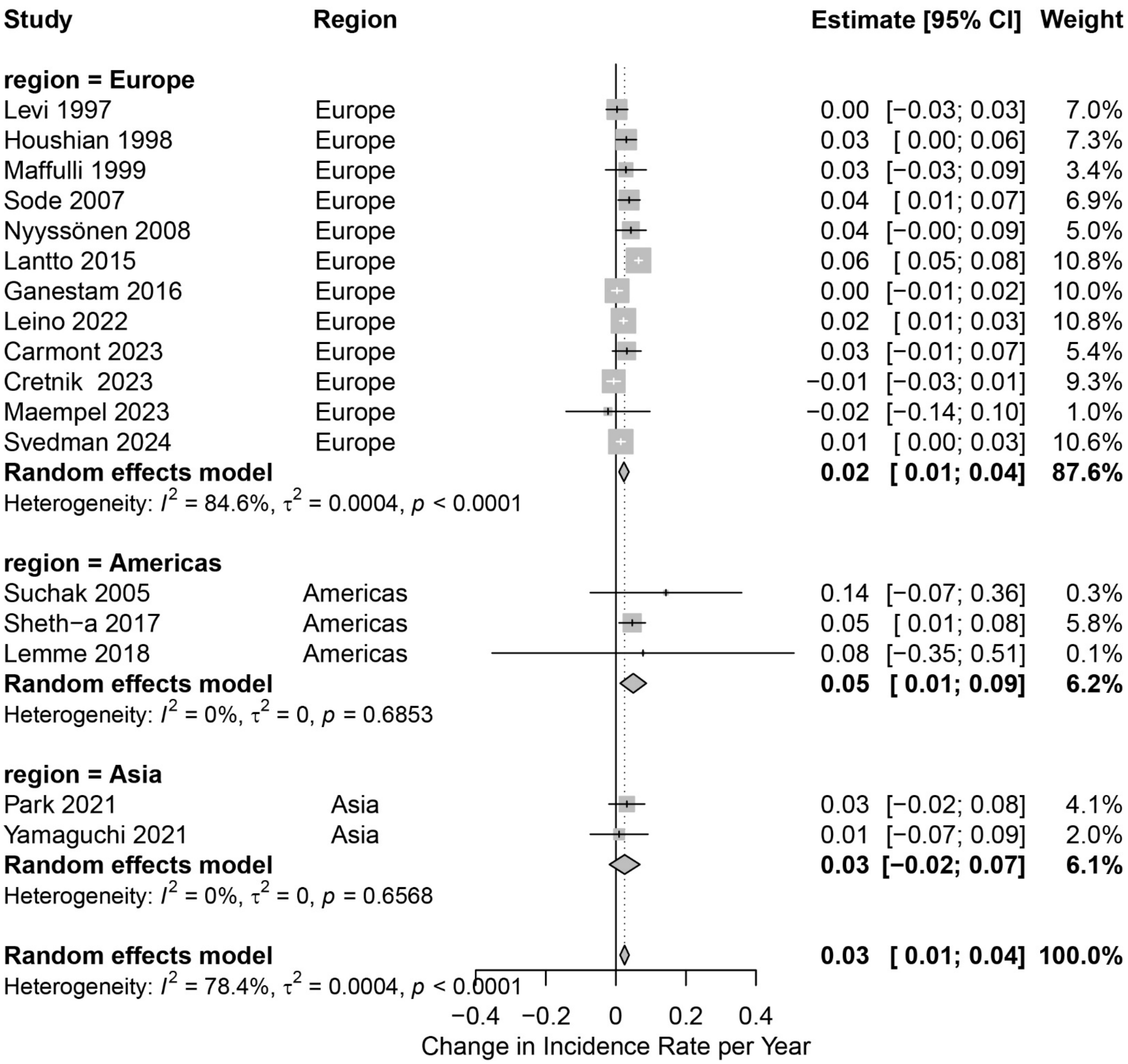

Fig. S6. Forest plot of temporal trends in Achilles tendon rupture incidence, stratified by continent. Within-study changes over time are displayed with 95% confidence intervals, and where applicable, pooled estimates are calculated for each region. Diamond markers denote the region-specific pooled incidence trends.

Individual studies temporal changes by sex

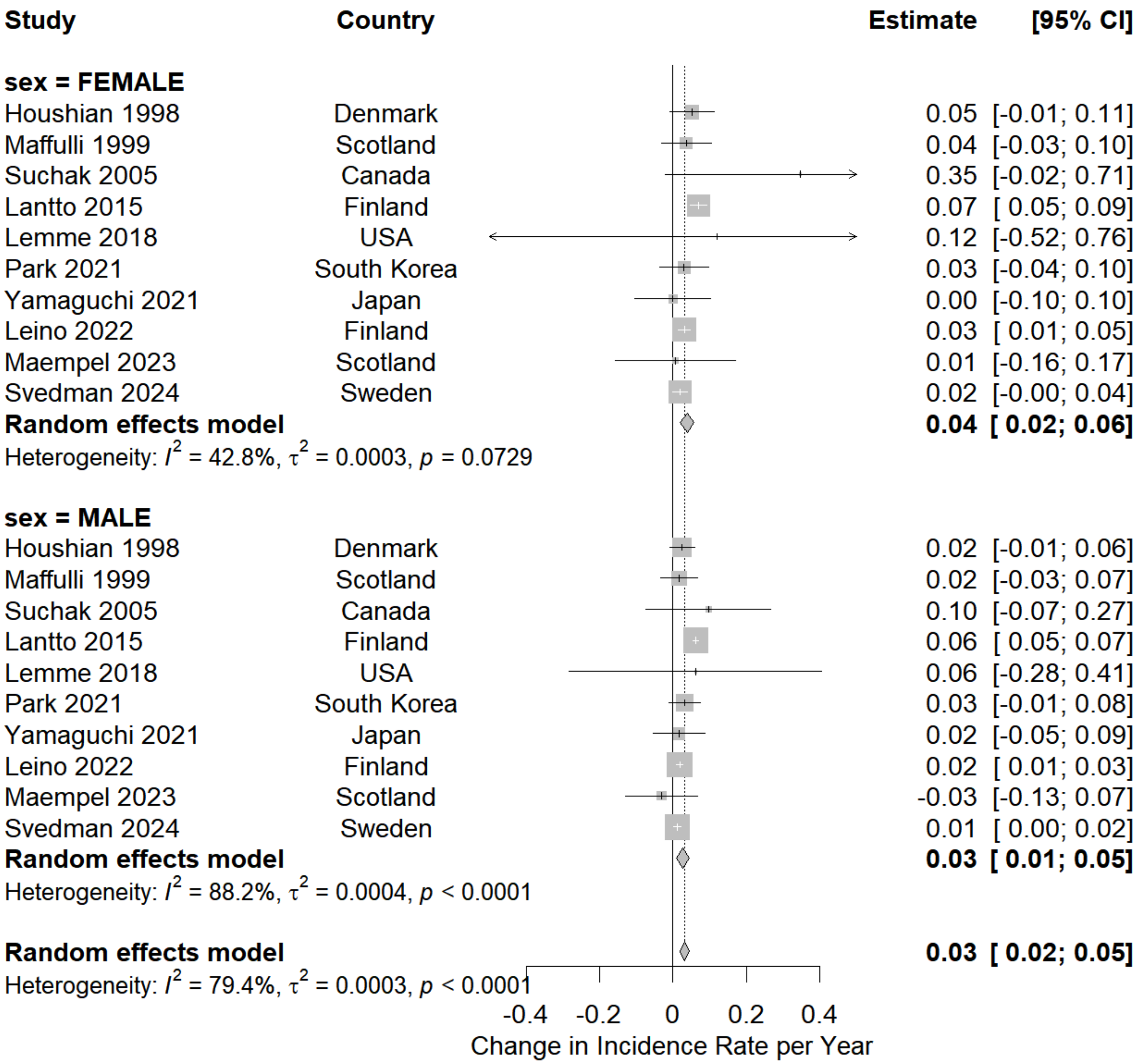

Fig. S7. Forest plot of temporal trends in Achilles tendon rupture incidence, stratified by sex. Within-study changes over time are displayed with 95% confidence intervals, and where applicable, pooled estimates are calculated for each country. Diamond markers denote the sex-specific pooled incidence trends.

Incidence rate of ATR by age group

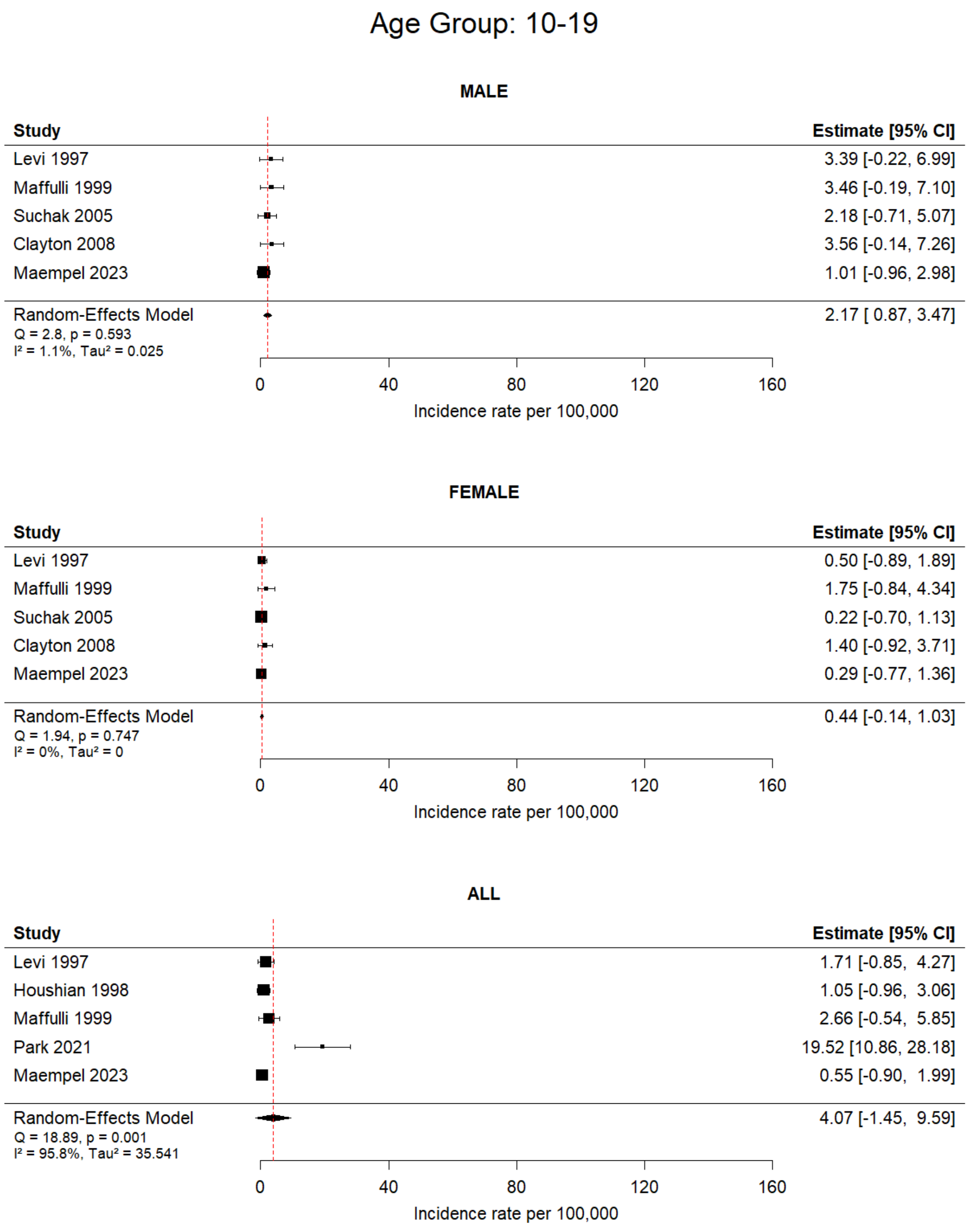

Fig. S8 Forest plots of incidence rates of Achilles tendon rupture per 100,000 person-years across age groups, stratified by sex. Each panel corresponds to one sex subgroup and presents the study-level incidence rates and 95% confidence intervals for a specific 10-year age group. The diamond represents the pooled incidence rate estimate from a random-effects model. The vertical dotted red line indicates the overall pooled estimate within each subgroup.

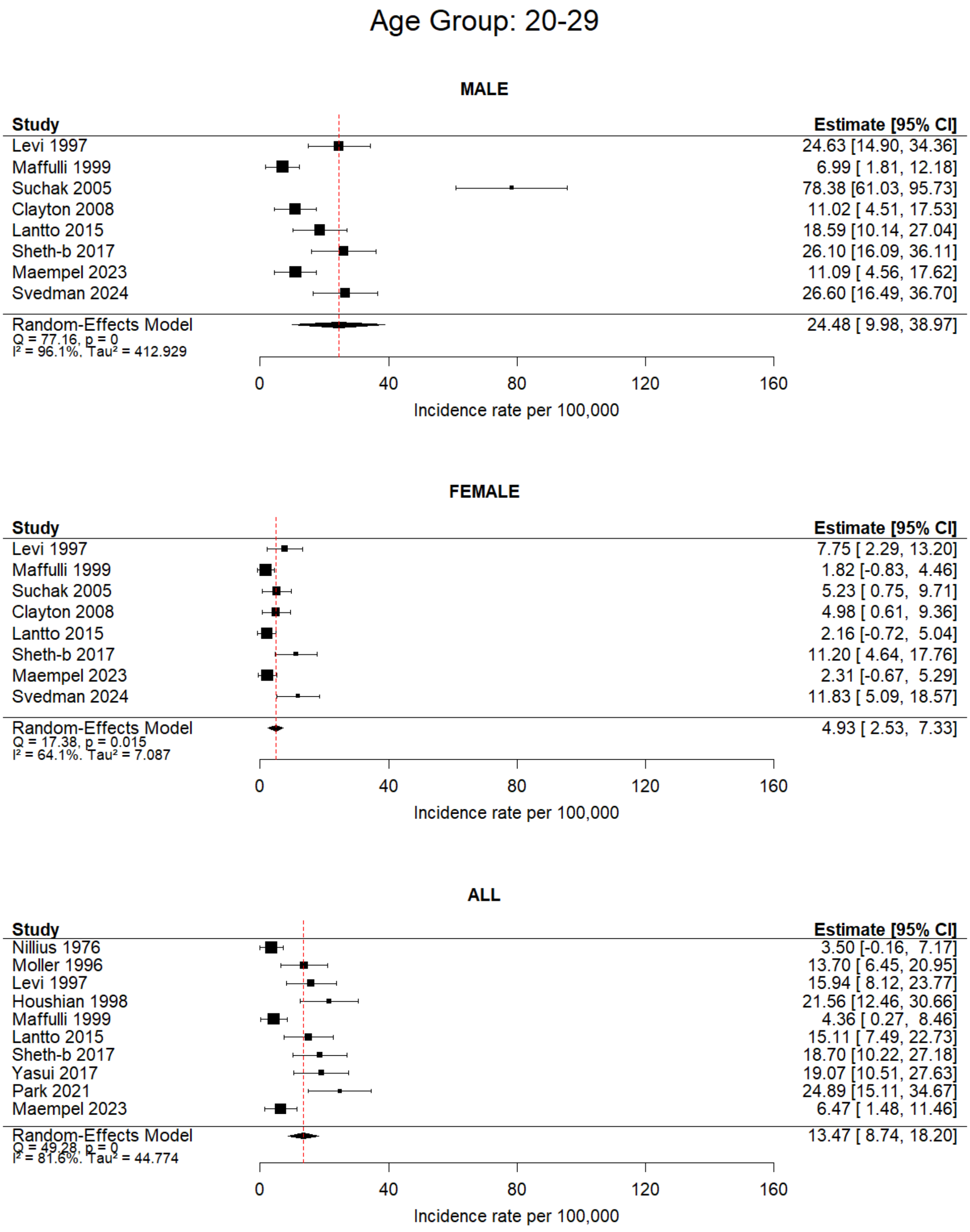

Fig. S9 Forest plots of incidence rates of Achilles tendon rupture per 100,000 person-years across age groups, stratified by sex. Each panel corresponds to one sex subgroup and presents the study-level incidence rates and 95% confidence intervals for a specific 10-year age group. The diamond represents the pooled incidence rate estimate from a random-effects model. The vertical dotted red line indicates the overall pooled estimate within each subgroup.

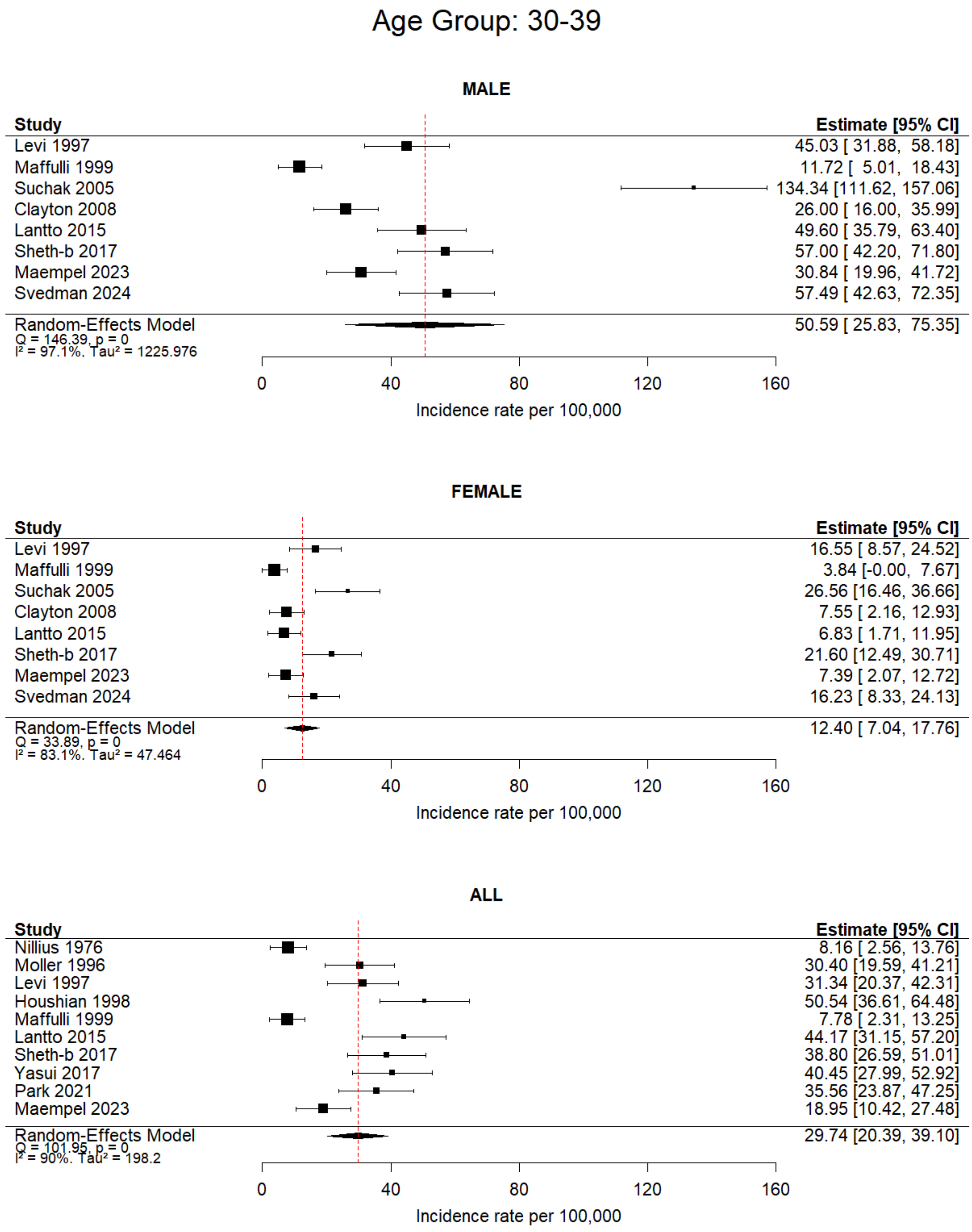

Fig. S10 Forest plots of incidence rates of Achilles tendon rupture per 100,000 person-years across age groups, stratified by sex. Each panel corresponds to one sex subgroup and presents the study-level incidence rates and 95% confidence intervals for a specific 10-year age group. The diamond represents the pooled incidence rate estimate from a random-effects model. The vertical dotted red line indicates the overall pooled estimate within each subgroup.

Age Group: 40-49

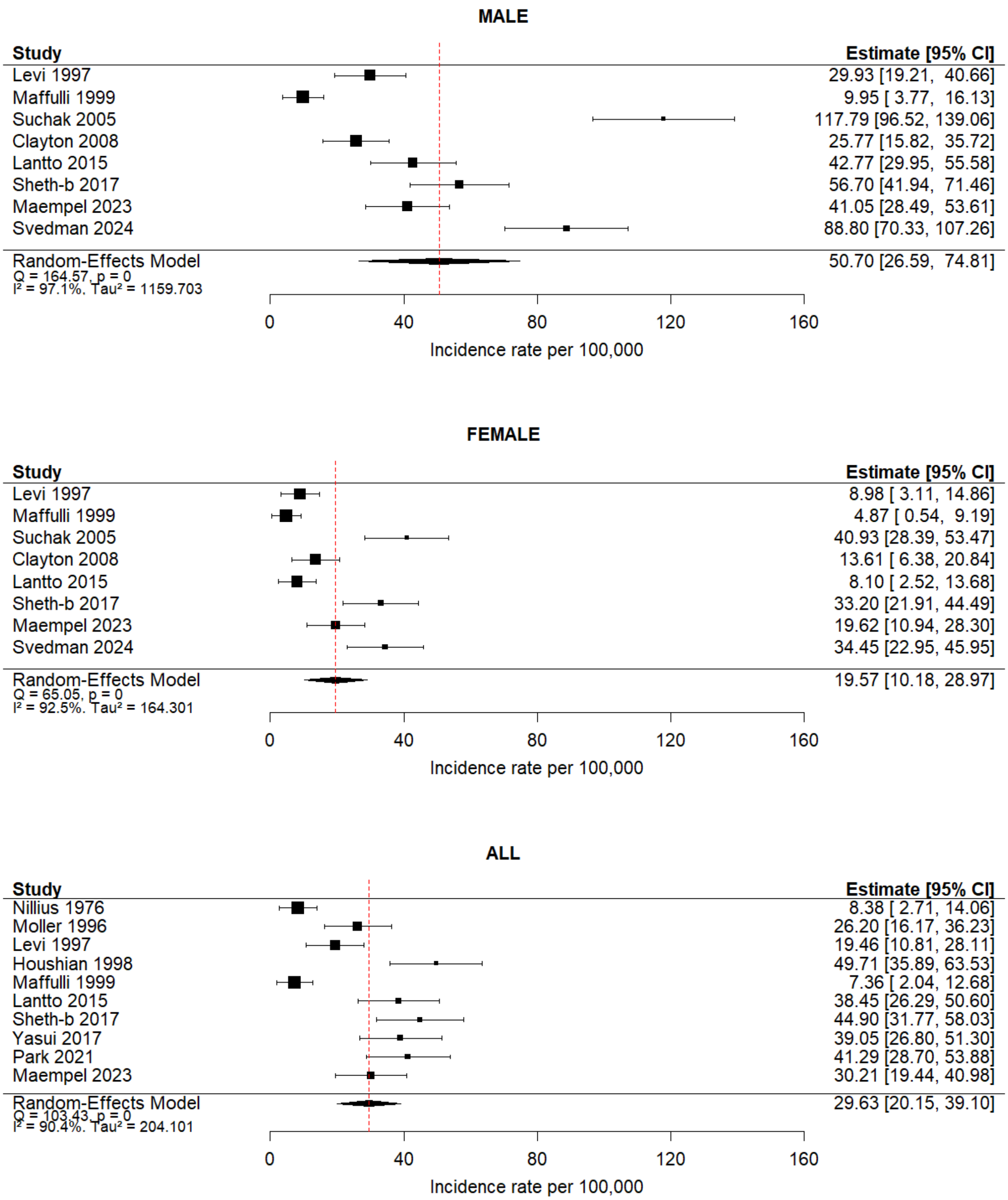

Fig. S11 Forest plots of incidence rates of Achilles tendon rupture per 100,000 person-years across age groups, stratified by sex. Each panel corresponds to one sex subgroup and presents the study-level incidence rates and 95% confidence intervals for a specific 10-year age group. The diamond represents the pooled incidence rate estimate from a random-effects model. The vertical dotted red line indicates the overall pooled estimate within each subgroup.

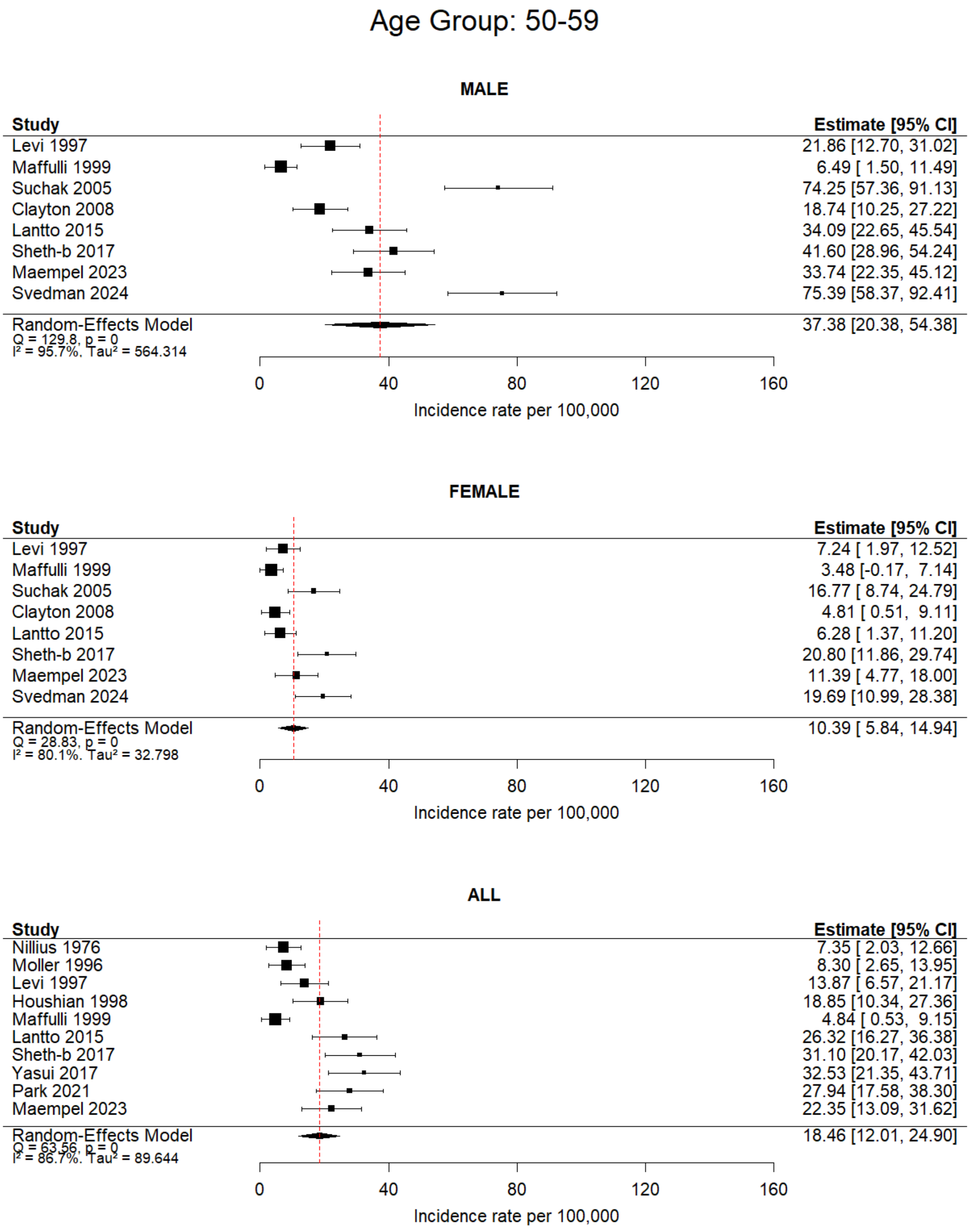

Fig. S12 Forest plots of incidence rates of Achilles tendon rupture per 100,000 person-years across age groups, stratified by sex. Each panel corresponds to one sex subgroup and presents the study-level incidence rates and 95% confidence intervals for a specific 10-year age group. The diamond represents the pooled incidence rate estimate from a random-effects model. The vertical dotted red line indicates the overall pooled estimate within each subgroup.

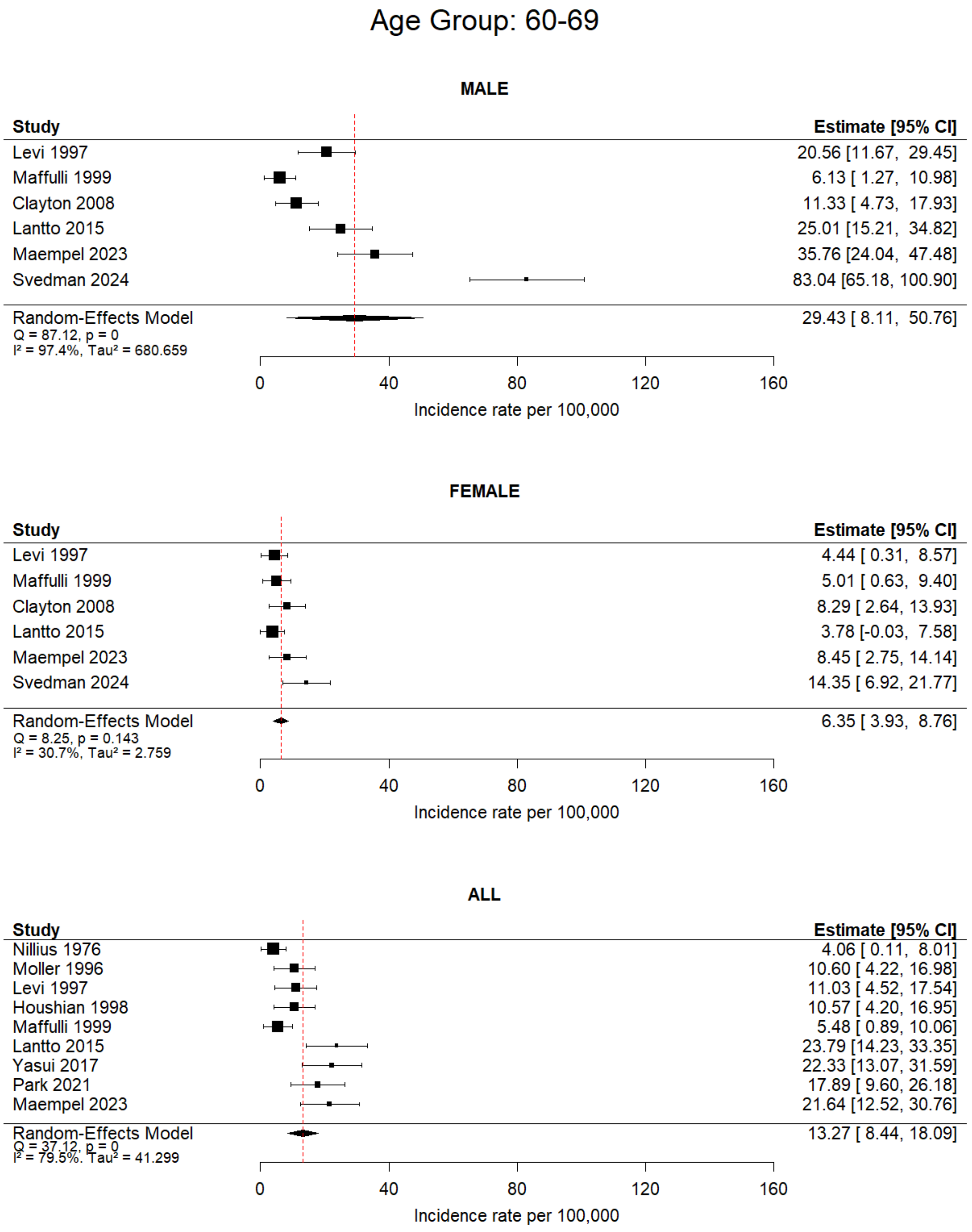

Fig. S13 Forest plots of incidence rates of Achilles tendon rupture per 100,000 person-years across age groups, stratified by sex. Each panel corresponds to one sex subgroup and presents the study-level incidence rates and 95% confidence intervals for a specific 10-year age group. The diamond represents the pooled incidence rate estimate from a random-effects model. The vertical dotted red line indicates the overall pooled estimate within each subgroup.

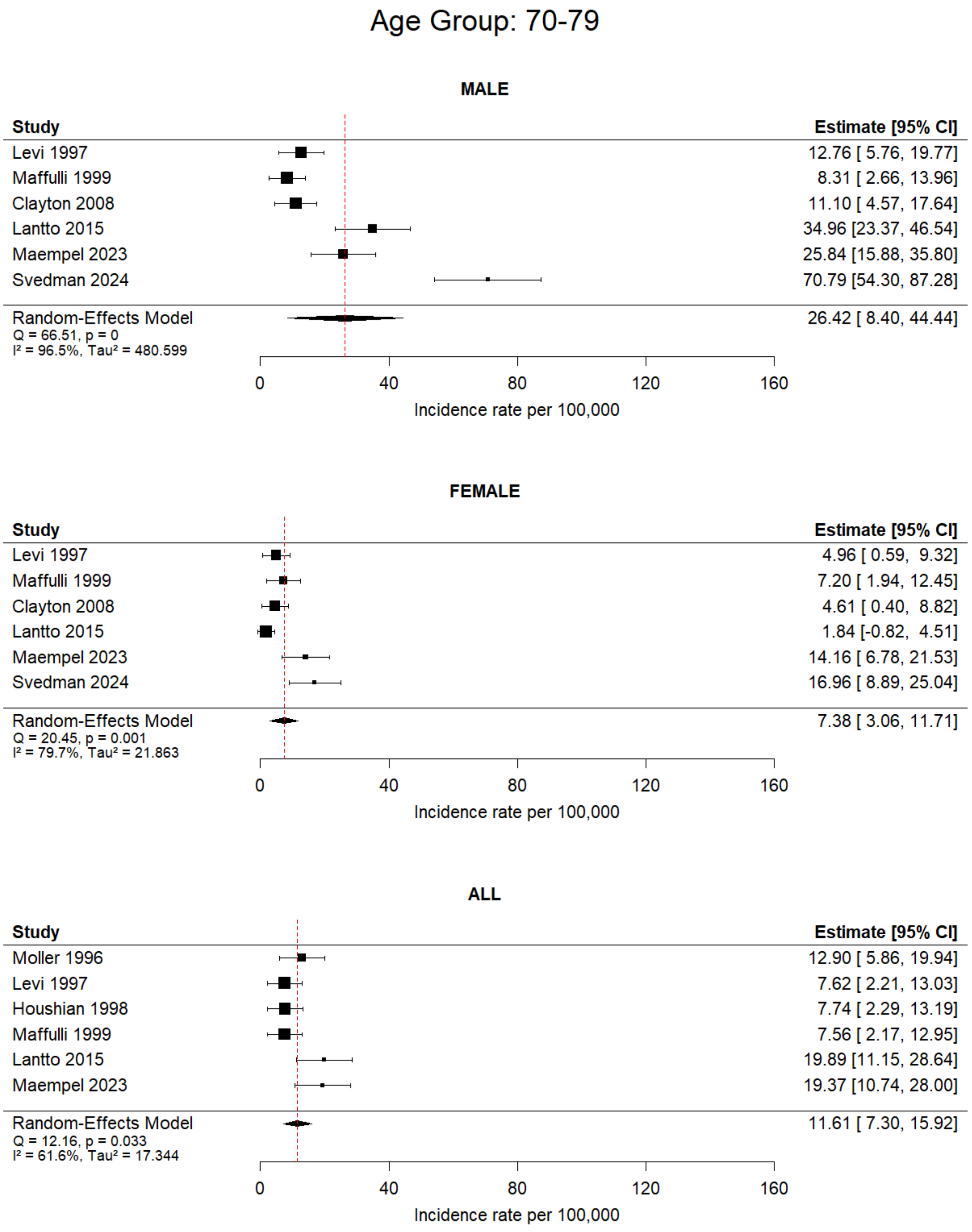

Fig. S14 Forest plots of incidence rates of Achilles tendon rupture per 100,000 person-years across age groups, stratified by sex. Each panel corresponds to one sex subgroup and presents the study-level incidence rates and 95% confidence intervals for a specific 10-year age group. The diamond represents the pooled incidence rate estimate from a random-effects model. The vertical dotted red line indicates the overall pooled estimate within each subgroup.

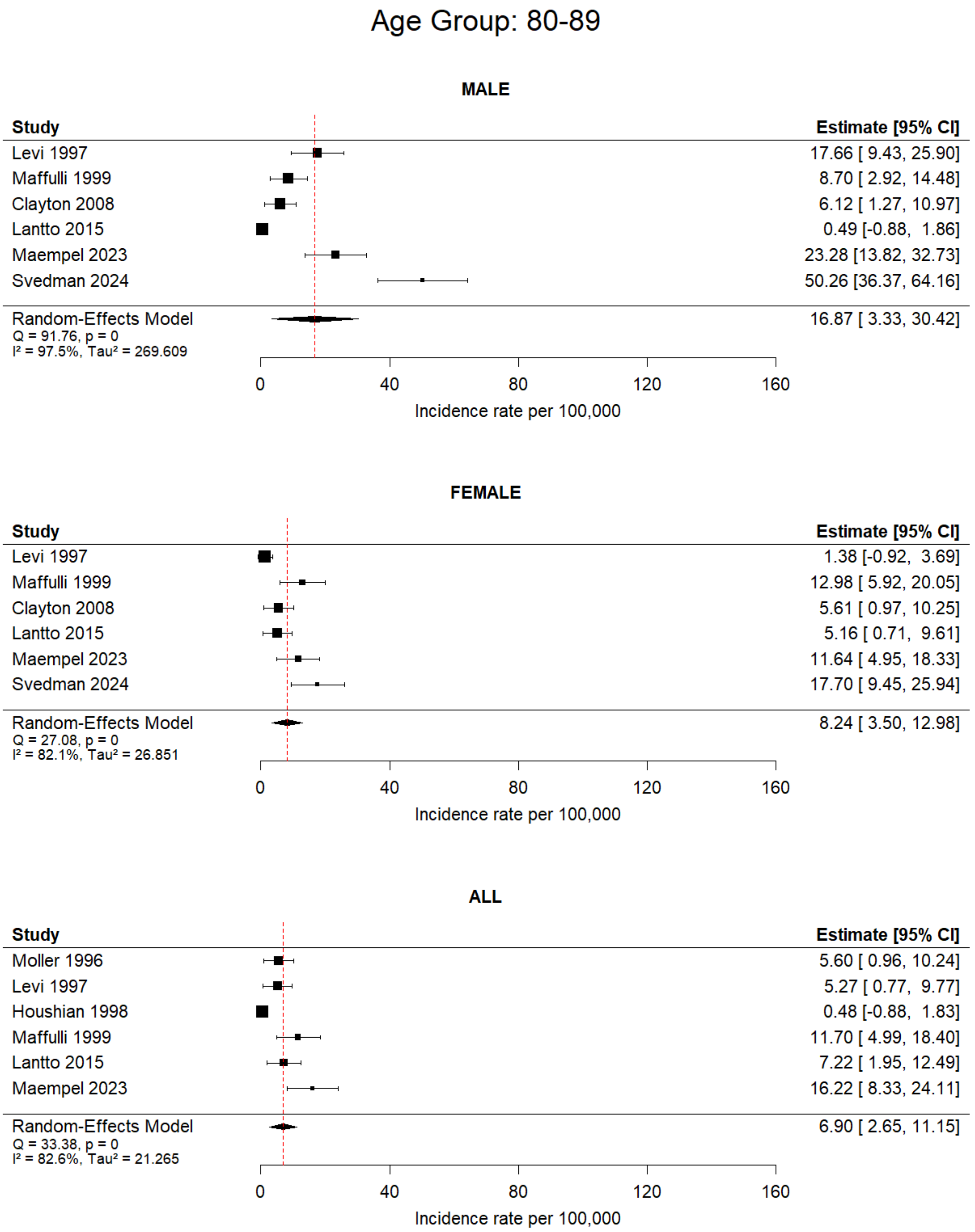

Fig. S15 Forest plots displaying pooled incidence rates of Achilles tendon rupture per 100,000 person-years across age groups, stratified by sex. Each panel corresponds to one sex subgroup and presents the study-level incidence rates and 95% confidence intervals for a specific 10-year age group. The diamond represents the pooled incidence rate estimate from a random-effects model. The vertical dotted red line indicates the overall pooled estimate within each subgroup.

Incidence rates by age group and sport participation

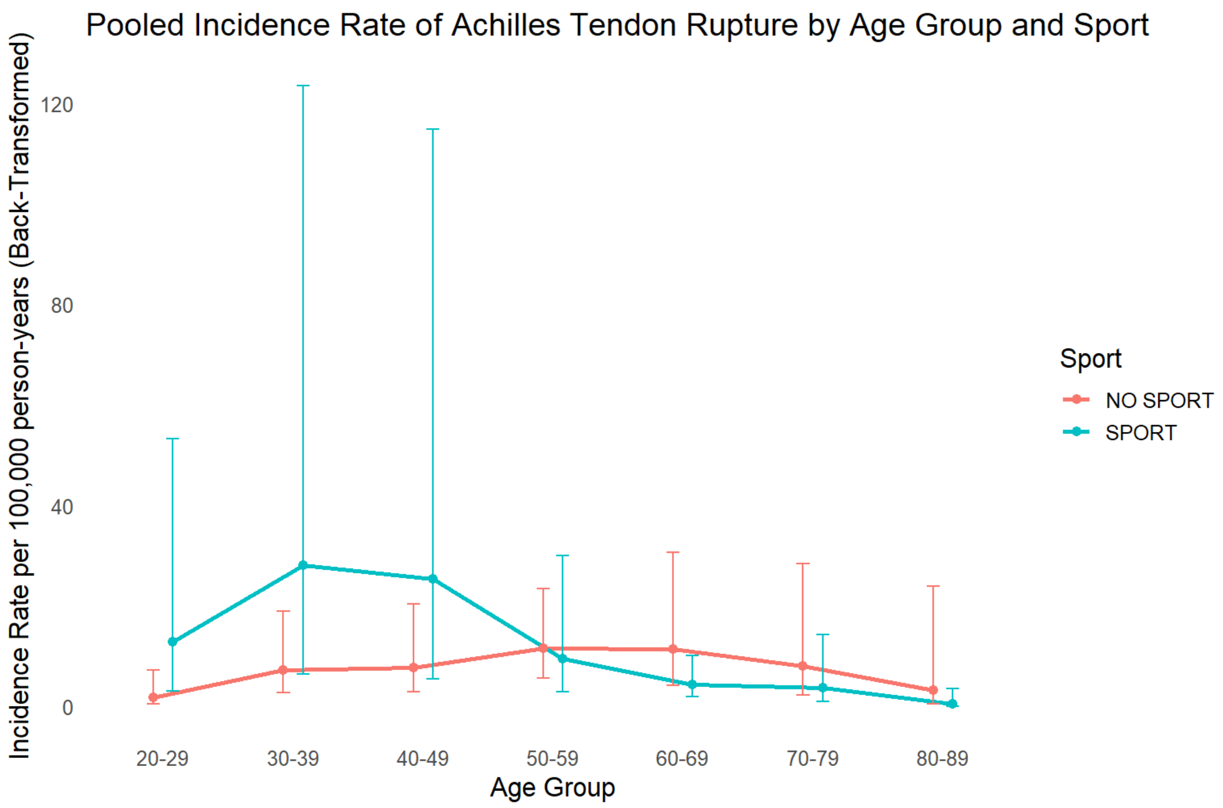

Fig. S16 Pooled incidence rates of Achilles tendon rupture per 100,000 person-years, stratified by age group and injury mechanism. Estimates are presented separately for sport-related (red) and non-sport-related (blue) ruptures. Rates were derived from a random-effects meta-analysis using log-transformed incidence values and inverse-variance weighting under Poisson assumptions, and are shown as back-transformed values with corresponding 95% confidence intervals.

References

1. Nillius SA, Nilsson BE, Westlin NE. The incidence of Achilles tendon rupture. Acta Orthop Scand. 1976 Feb;47(1):118-21.
2. Moller A, Astrom M, Westlin NE. Increasing incidence of Achilles tendon rupture. Acta Orthopaedica Scandinavica. 1996;67(5):479-81.
3. Levi N. The incidence of Achilles tendon rupture in Copenhagen. Injury. 1997;28(4):311-3.
4. Houshian S, Tscherning T, Riegels-Nielsen P. The epidemiology of achilles tendon rupture in a Danish county. Injury. 1998;29(9):651-4.
5. Maffulli N, Waterston SW, Squair J, Reaper J, Douglas AS. Changing incidence of Achilles tendon rupture in Scotland: a 15-year study. Clinical journal of sport medicine : official journal of the Canadian Academy of Sport Medicine. 1999;9(3):157-60.
6. Suchak AA, Bostick G, Reid D, Blitz S, Jomha N. The incidence of Achilles tendon ruptures in Edmonton, Canada. Foot and Ankle International. 2005;26(11):932-6.
7. Sode J, Obel N, Hallas J, Lassen A. Use of fluroquinolone and risk of Achilles tendon rupture: A population-based cohort study. European Journal of Clinical Pharmacology. 2007;63(5):499-503.
8. Tumilty S. Achilles tendon rupture: rising incidence in New Zealand follows international trends. Physical Therapy Reviews. 2007;12(1):59-65.
9. Clayton RAE, Court-Brown CM. The epidemiology of musculoskeletal tendinous and ligamentous injuries. Injury. 2008;39(12):1338-44.
10. Nyyssonen T, Luthje P, Kroger H. The increasing incidence and difference in sex distribution of Achilles tendon rupture in Finland in 1987-1999. Scandinavian Journal of Surgery. 2008;97(3):272-5.
11. Gwynne-Jones DP, Sims M, Handcock D. Epidemiology and outcomes of acute achilles tendon rupture with operative or nonoperative treatment using an identical functional bracing protocol. Foot and Ankle International. 2011;32(4):337-43.
12. Lantto I, Heikkinen J, Flinkkilä T, Ohtonen P, Leppilahti J. Epidemiology of Achilles tendon ruptures: increasing incidence over a 33-year period. Scand J Med Sci Sports. 2015 Feb;25(1):133-8.
13. Mattila VM, Huttunen TT, Haapasalo H, Sillanpaa P, Malmivaara A, Pihlajamaki H. Declining incidence of surgery for Achilles tendon rupture follows publication of major RCTs: evidence-influenced change evident using the Finnish registry study. British journal of sports medicine. 2015;49(16):1084-6.
14. Ganestam A, Kallemose T, Troelsen A, Barfod KW. Increasing incidence of acute Achilles tendon rupture and a noticeable decline in surgical treatment from 1994 to 2013. A nationwide registry study of 33,160 patients. Knee surgery, sports traumatology, arthroscopy : official journal of the ESSKA. 2016;24(12):3730-7.
15. Sheth U, Moineddin R, Jaglal S, Wasserstein D, Jenkinson R, Kreder H. Practice patterns in the care of acute Achilles tendon ruptures: Is there an association with level I evidence? Bone and Joint Journal. 2017;99B(12):1629-36.
16. Sheth U, Wasserstein D, Jenkinson R, Moineddin R, Kreder H, Jaglal SB. The epidemiology and trends in management of acute Achilles tendon ruptures in Ontario, Canada: a population-based study of 27 607 patients. The bone & joint journal. 2017;99-B(1):78-86.
17. Yasui Y, Tonogai I, Rosenbaum AJ, Shimozono Y, Kawano H, Kennedy JG. The Risk of Achilles Tendon Rupture in the Patients with Achilles Tendinopathy: Healthcare Database Analysis in the United States. BioMed Research International. 2017;2017:7021862.

18. Lemme NJ, Li NY, DeFroda SF, Kleiner J, Owens BD. Epidemiology of Achilles Tendon Ruptures in the United States: Athletic and Nonathletic Injuries From 2012 to 2016. Orthopaedic Journal of Sports Medicine. 2018;6(11).
19. Longo U, Salvatore G, Risi Ambrogioni L, Cella E, Candela V, Carnevale A, et al. Epidemiology of Achilles tendon surgery in Italy: a nationwide registry study, from 2001 through 2015. BMC Musculoskeletal Disorders. 2020;21(1):687.
20. Park HG, Youn D, Baik JM, Hwang JH. Epidemiology of Achilles Tendon Rupture in South Korea: Claims Data of the National Health Insurance Service from 2009 to 2017. CiOS Clinics in Orthopedic Surgery. 2021;13(4):539-48.
21. Yamaguchi S, Kimura S, Akagi R, Yoshimura K, Kawasaki Y, Shiko Y, et al. Increase in Achilles Tendon Rupture Surgery in Japan: Results From a Nationwide Health Care Database. Orthopaedic Journal of Sports Medicine. 2021;9(10).
22. Leino O, Keskinen H, Laaksonen I, Makela K, Loyttyniemi E, Ekman E. Incidence and Treatment Trends of Achilles Tendon Ruptures in Finland: A Nationwide Study. Orthopaedic Journal of Sports Medicine. 2022;10(11).
23. Park KH, Park JH, Yoon YK, Kwon JB, Kim JH, Lee E, et al. Association between outdoor temperature and achilles tendon repair: A 14-years nationwide population-based cohort study. PLoS ONE. 2022;17(3 March):e0265041.
24. Carmont MR, Morgan F, Fakoya K, Heaver C, Brorsson A, Nilsson-Helander K. The influence of the COVID pandemic on the epidemiology of Achilles tendon ruptures in east Shropshire, United Kingdom. Journal of ISAKOS. 2023;8(2):94-100.
25. Cretnik A, Kosir R. Incidence of Achilles tendon rupture: 25-year regional analysis with a focus on bilateral ruptures. Journal of International Medical Research. 2023;51(11).
26. Maempel JF, Clement ND, Mackenzie SP, McCann C, White TO. Socioeconomic deprivation status predicts both the incidence and nature of Achilles tendon rupture. Knee surgery, sports traumatology, arthroscopy : official journal of the ESSKA. 2023;31(2):691-700.
27. Briggs-Price S, Mangwani J, Houchen-Wolloff L, Modha G, Fitzpatrick E, Faizi M, et al. Incidence, demographics, characteristics and management of acute Achilles tendon rupture: An epidemiological study. PLoS ONE. 2024;19(6 June):e0304197.
28. Svedman S, Marcano A, Ackermann PW, Fellander-Tsai L, Berg HE. Acute Achilles tendon ruptures between 2002-2021: sustained increased incidence, surgical decline and prolonged delay to surgery - a nationwide study of 53 688 ruptures in Sweden. BMJ Open Sport and Exercise Medicine. 2024;10(3):e001960.
